# From routine full-spine radiographs to decision-oriented Risser stratification: an interpretable deep-learning approach

**DOI:** 10.3389/fped.2026.1832066

**Published:** 2026-06-17

**Authors:** Zexi Wang, Yuan Zhang, Yixi Wang, Feng Xue, Yi Yang, Wen Zhao

**Affiliations:** 1 Department of Minimally Invasive Spine and Precision Orthopedics, The First Affiliated Hospital of Xinjiang Medical University, Urumqi, China; 2 Xinjiang Medical-Engineering Integration Technology Center, The First Affiliated Hospital of Xinjiang Medical University, Urumqi, China

**Keywords:** adolescent idiopathic scoliosis (AIS), binary classification, clinical decision support, deep learning, Grad-CAM, ResNet-18, risser staging, skeletal maturity assessment

## Abstract

**Background:**

Accurate assessment of remaining growth is crucial for risk stratification and treatment planning in adolescent idiopathic scoliosis (AIS). Risser staging is widely used but demonstrates only moderate reproducibility in routine practice, particularly on standing full-spine radiographs where the iliac apophysis occupies a small area. This study aimed to develop and evaluate an interpretable, decision-oriented deep-learning model for automatic stratification of skeletal maturity as Risser 0–2 vs. 3–5 using routine full-spine radiographs.

**Methods:**

This single-center retrospective study included 875 standing posteroanterior full-spine radiographs from AIS patients aged 10–18 years. An expert consensus reference standard was established using a three-surgeon adjudication process. A predefined, rule-based pelvic region of interest was automatically extracted for model training. The data were split at the patient level into training, validation, and test sets (70%/10%/20%, stratified). Multiple convolutional neural network architectures were evaluated, and ResNet-18 was selected based on performance stability. Model interpretability was assessed using gradient-weighted class activation mapping (Grad-CAM). An independent clinical reader study was conducted on 50 sampled test cases, in which three spine surgeons completed two reading sessions (without and with model assistance) to assess agreement with the expert reference and reading time.

**Results:**

For binary stratification (Risser 0–2 vs. 3–5), the selected model achieved an area under the receiver operating characteristic curve (AUC) of 0.938, an accuracy of 0.875, and a Cohen's kappa of 0.748 on the independent test set. Grad-CAM visualizations indicated that model predictions focused on the iliac apophysis and adjacent ossification regions. In the reader study, compared with unaided reading, model assistance reduced the mean reading time by 9.7–11.8 s per case across the three readers, from 34.7–43.2 s to 25.0–31.8 s (all *P* < 0.001) and improved agreement with the expert reference, with the most pronounced gains observed among junior surgeons. Exploratory six-class Risser staging showed substantially lower performance, particularly for intermediate stages.

**Conclusions:**

Using routine standing full-spine radiographs, an interpretable deep-learning model enabled clinically actionable Risser stratification and improved reading efficiency in an independent reader study, without the need for additional imaging. This approach may support the standardized assessment of growth potential in routine AIS management.

## 1 Introduction

AIS is one of the most common coronal spinal deformities seen in adolescents without identifiable underlying disease (1). Curve progression is closely related to remaining growth, and clinical management strategies (2, 3)—including observation, bracing, and surgery—are determined not only by curve magnitude but also by skeletal maturity in routine practice (4–6). Reliable assessment of residual growth potential is therefore essential for risk stratification and treatment planning in patients with AIS (2).

Risser staging is based on the degree of ossification and fusion of the iliac apophysis as seen on pelvic or full-spine radiographs, and is widely used in clinical practice as a surrogate marker of skeletal maturity (7–9). Lower Risser stages (0–2) generally indicate a period of rapid growth and a higher risk of curve progression, whereas higher stages (3–5) usually suggest that skeletal maturity is approaching or has been reached, with a substantially reduced likelihood of further progression (2, 6). In routine clinical practice, Risser staging is commonly interpreted in conjunction with the Cobb angle and other maturity indicators to guide follow-up intervals, duration of brace treatment, and timing of surgical intervention (4–6). The relationship between iliac crest maturation and skeletal as well as chronological age has also been described in previous studies (10). However, skeletal maturation does not occur at the same pace in all adolescents. Sex-related differences in pubertal timing and population-level differences related to ethnic background may affect how a given Risser stage reflects remaining growth. These factors are important when considering the external validity of any Risser-based prediction or decision-support model (11).

Although Risser staging is widely adopted in clinical practice, manual assessment has notable limitations. Previous studies have reported only moderate inter- and intra-observer agreement, indicating substantial variability both between different readers and within the same reader over time when visually determining the onset and completion of iliac apophyseal ossification (12, 13). In addition, image quality, pelvic rotation, and overlap from bowel gas or soft tissues may further obscure the iliac crest, while the iliac apophysis occupies only a small portion of standing full-spine radiographs. Similar concerns regarding the inter- and intra-observer reliability of the Risser sign, particularly with respect to variability among readers, have also been reported in the literature (14).

For supervised model development, this subjectivity is not only a clinical reading issue but also a potential source of reference-label noise. In Risser staging, adjacent stages are not always visually discrete, especially when the iliac apophysis is faint, partially obscured, or affected by pelvic rotation. Disagreement between readers may therefore reflect not only individual reader error, but also the intrinsic ambiguity of the radiographic reference standard itself. This issue is particularly relevant for fine-grained six-stage classification, where small visual differences between neighboring stages may be difficult to define consistently on routine full-spine radiographs. Together, these factors make Risser interpretation prone to subjectivity and increase the workload in high-volume spine clinics.

With advances in deep learning, automated assessment of skeletal maturity from medical images has attracted increasing attention. Convolutional neural network (CNN)–based systems have demonstrated promising performance in bone age assessment and other maturity-related tasks (15–17). More recently, several studies have developed models capable of predicting Risser stage directly from routine pelvic radiographs or abdominal x-ray images, achieving accuracy comparable to that of expert readers and suggesting potential value as decision-support tools (18, 19). Other approaches have employed pelvis-centered network architectures or multi-task frameworks to jointly perform iliac region localization and six-stage Risser classification (18, 20). In addition, machine-learning methods based on EOS whole-body imaging have been proposed to estimate Risser stage and spinal growth potential (21). Collectively, these studies indicate that automated Risser assessment is feasible under controlled imaging conditions and can reach expert-level performance.

However, most existing automated Risser staging models have been trained on dedicated pelvic radiographs or EOS biplanar images, rather than on routine standing full-spine digital radiographs obtained during AIS follow-up (18, 19, 21). In addition, prior studies have predominantly focused on six-grade Risser classification, while comparatively little attention has been paid to the clinically relevant binary stratification (Risser 0–2 vs. 3–5) (6). This dichotomization directly reflects remaining growth potential and represents a key reference for bracing decisions and surgical timing. Finally, although segmentation-based approaches or Grad-CAM visualizations have been used to improve model interpretability, it remains unclear whether the decision basis of these models on full-spine radiographs truly aligns with the iliac apophysis–centered anatomical features relied upon by clinicians in routine practice (22).

Based on these considerations, this study aimed to develop and evaluate a clinically oriented approach for automated skeletal maturity stratification using standing posteroanterior full-spine radiographs. The primary focus was to distinguish patients with Risser stages 0–2 from those with stages 3–5, thereby reflecting whether meaningful growth potential remains and providing a practical reference for follow-up scheduling, brace management, and surgical timing. Compared with full six-stage Risser classification, this binary grouping may also be less affected by the ambiguity between adjacent Risser stages, which is a common source of variability in manual assessment.

To this end, we adopted a standardized pelvic region-of-interest (ROI) cropping strategy to focus the model on the iliac apophysis and trained convolutional neural networks to perform the above binary classification task. Model interpretability was further assessed using gradient-weighted class activation mapping (Grad-CAM) to examine whether the regions driving model predictions were consistent with the anatomical features relied upon in clinical assessment. In addition, as a supplementary analysis, we evaluated performance in six-grade Risser classification to illustrate the inherent difficulty of fine-stage discrimination on routine standing full-spine radiographs.

## 2 Materials and methods

### 2.1 Study population and image acquisition

This retrospective study included patients who presented to our institution with AIS and underwent standing posteroanterior full-spine digital radiography. A total of 1,119 radiographic examinations were initially identified.

Inclusion criteria were as follows: (1) a confirmed diagnosis of AIS; (2) age between 10 and 18 years; (3) baseline standing posteroanterior full-spine radiographs with clear visualization of the pelvic region.

Exclusion criteria included: (1) a history of prior spinal surgery; (2) non-baseline examinations, such as follow-up radiographs; (3) inadequate image quality, such as marked overexposure, underexposure, or motion artifacts; (4) incomplete visualization or occlusion of the pelvic contour or iliac crest region; and (5) scoliosis secondary to congenital, neuromuscular, or syndromic conditions based on clinical records.

After applying these criteria, a total of 875 radiographic images were included in the final analysis. The study protocol was approved by the institutional ethics committee and conducted in accordance with the Declaration of Helsinki. Owing to the retrospective design of the study and the use of fully anonymized data, the requirement for informed consent was waived by the ethics committee.

### 2.2 Image preprocessing and pelvic ROI extraction

All radiographic images were exported in digital format and underwent standardized preprocessing. To reduce interference from irrelevant anatomical structures such as the thorax, lumbar spine, and proximal femora, and to focus the model on the iliac apophysis that is most relevant for Risser staging, a standardized pelvic ROI was automatically extracted from each standing full-spine radiograph. Specifically, the central 60% of the image width and the inferior 35% of the image height were retained. This rule was designed to include the bilateral iliac crests and iliac apophyseal regions, which are the key anatomical structures for Risser assessment.

This rule-based cropping strategy was verified on randomly sampled cases and was able to consistently cover the bilateral iliac crests (Supplementary Figure S1). Because marked scoliosis-related rotation, pelvic obliquity, or asymmetric pelvic positioning may shift the iliac crests away from a fixed crop, images in which the bilateral iliac crests or pelvic contour could not be reliably included were excluded during the quality control process.

The cropped ROI images were directly resized to 384 × 384 pixels to provide a fixed input size for CNN training and inference. To accommodate convolutional neural networks initialized with ImageNet pre-trained weights, images were converted to three-channel format and normalized using a standardized preprocessing pipeline before being used for model training and inference.

**Figure 1:**
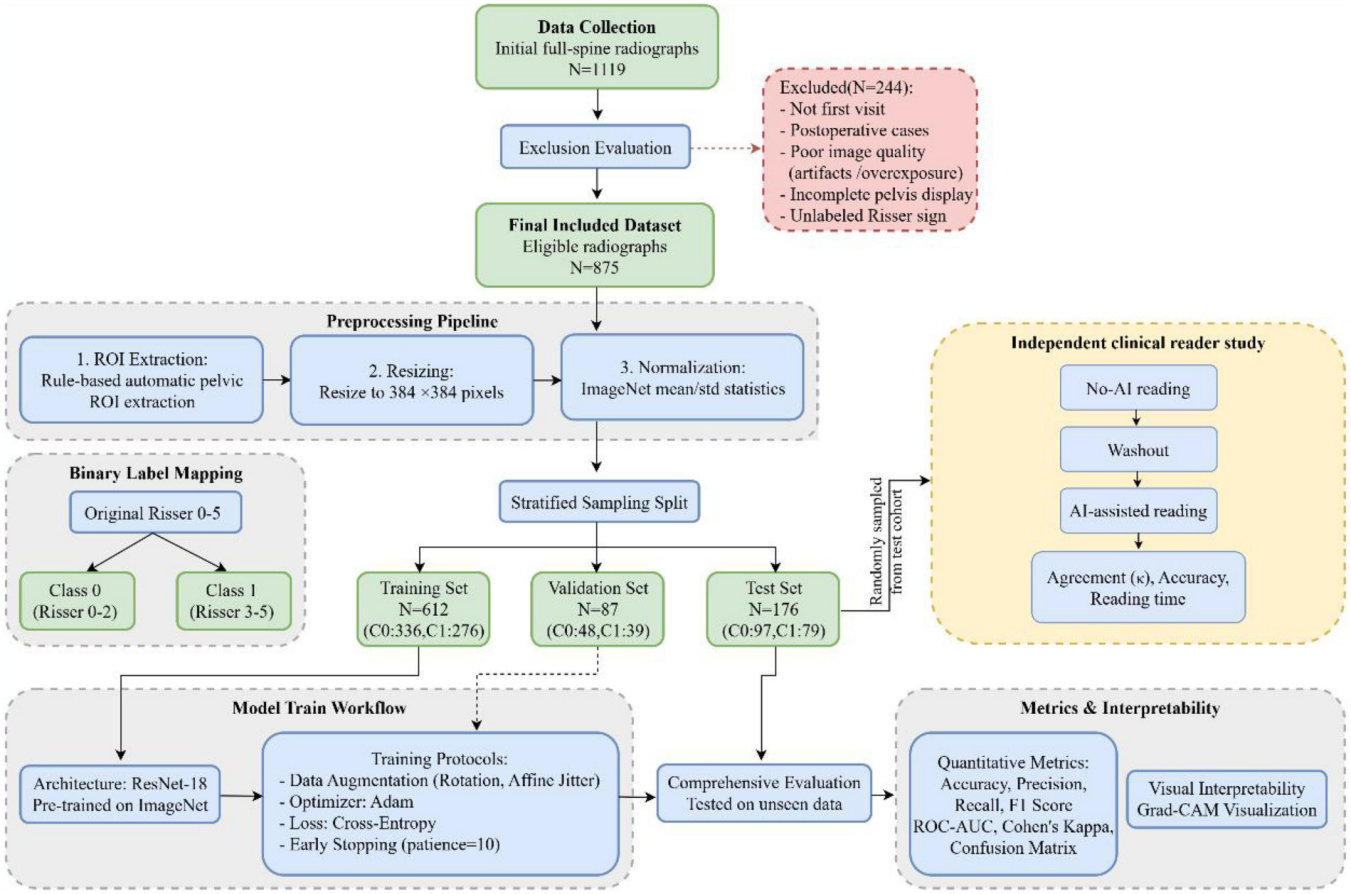
Overview of the study workflow. The figure illustrates the overall design of the study, including patient selection, preprocessing of standing full-spine radiographs, construction of clinically relevant binary labels, stratified data splitting, model training, and performance evaluation. Key steps include automated pelvic ROI extraction, standardized image preprocessing, and training of a convolutional neural network (ResNet-18) to predict skeletal maturity (Risser 0–2 vs. 3–5). In parallel, an independent clinical reader study was conducted on a randomly sampled subset of the test cohort to evaluate the effect of AI assistance on reader agreement with the expert reference standard, diagnostic accuracy, and reading time. Model performance and clinical relevance were further assessed using quantitative metrics and Grad-CAM-based visual interpretability.

It should be noted that the above ROI extraction and preprocessing procedures were applied only during the model training and inference phases. In the clinical reader study, all readers performed assessments based on the original standing full-spine radiographs to closely reflect real-world clinical reading conditions. An overview of the study workflow, including case selection, image preprocessing, model development, and evaluation, is shown in Figure 1.

### 2.3 Risser grading and reference standard

Risser staging for all patients was performed by three spine surgeons with dedicated experience in spine surgery. Each standing posteroanterior full-spine radiograph was initially assessed by an attending spine surgeon with 10 years of experience. All images were then independently reviewed by a chief spine surgeon with 20 years of experience, who was blinded to the initial assessment.

For cases with discrepant ratings between the first two readers, another chief spine surgeon with 30 years of experience participated in adjudication. Final Risser grades were determined through consensus discussion among the involved surgeons. This expert consensus grading was used as the reference standard for model training and performance evaluation. This workflow was intended to reduce subjective reference-label variability, although residual ambiguity in borderline Risser stages could not be fully eliminated.

### 2.4 Classification task definition

The primary task of this study was a binary classification designed to address clinically relevant decision-making. Specifically, Risser stages 0–2 were grouped as skeletally immature, whereas Risser stages 3–5 were grouped as near or fully mature, reflecting differences in remaining growth potential and the associated risk of curve progression in patients with adolescent idiopathic scoliosis.

In addition, an exploratory analysis was performed using the original six-grade Risser classification (stages 0–5). This supplementary analysis aimed to illustrate the inherent difficulty of fine-grained discrimination between adjacent Risser stages when using routine standing full-spine radiographs, as well as the typical patterns of misclassification observed under these imaging conditions.

### 2.5 Data partitioning

The dataset was partitioned at the patient level using a stratified sampling strategy to preserve the distribution of the two Risser groups across subsets. The full cohort was divided into a training set (*n* = 612), a validation set (*n* = 87), and an independent test set (*n* = 176).

### 2.6 Deep-learning model development

Three widely used CNN architectures—ResNet-18, ResNet-34, and DenseNet-121—were selected as candidate models for classification (23, 24). All networks were initialized with ImageNet pre-trained weights, and the final classification layers were replaced to match the output requirements of the present task.

The same training protocol was applied across all candidate CNN architectures to ensure a fair comparison. The training configuration is summarized in Supplementary Table S1. Briefly, models were trained using cross-entropy loss and the Adam optimizer with an initial learning rate of 1 × 10⁻^4^ and a batch size of 4. The maximum number of training epochs was set to 50. Early stopping was applied when validation accuracy did not improve for 10 consecutive epochs, and the model with the highest validation accuracy was saved for evaluation. A fixed random seed of 42 was used for data splitting and model training.

During training, data augmentation included random rotation within ±7°, random affine translation up to 2% in both directions, random scaling from 0.95 to 1.05, and brightness and contrast jittering within ±0.1. Validation and test images were not augmented. All images were normalized using the ImageNet mean and standard deviation before being input into the networks.

**Figure 2:**
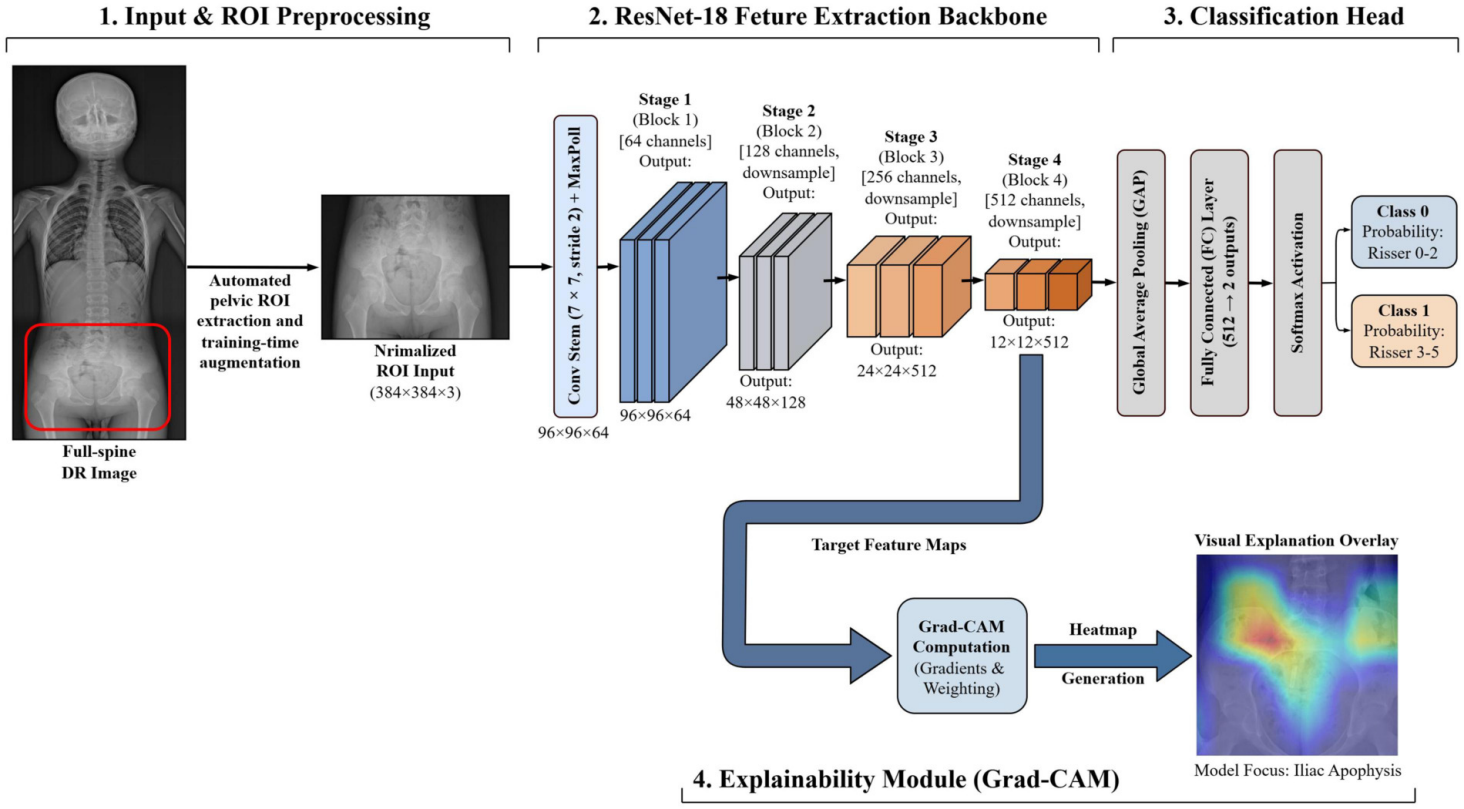
Architecture of the proposed deep-learning model for automated Risser staging. The model consists of four main components: (1) Input preprocessing, where the pelvic region is automatically cropped from the full-spine radiograph and normalized to a fixed resolution; (2) Feature extraction, performed by a ResNet-18 backbone with progressively deeper convolutional stages; (3) Binary classification head that outputs the probability of early (Risser 0–2) or advanced (Risser 3–5) skeletal maturity; and (4) Explainability module, where Grad-CAM highlights discriminative regions—primarily the iliac apophysis—to provide visual interpretability aligned with clinical assessment.

Based on subsequent performance evaluation, ResNet-18 was selected for further analyses. The overall architecture of the selected model is illustrated in Figure 2.

### 2.7 Model evaluation and interpretability

For both the binary and six-class classification tasks, overall accuracy, precision, recall, F1 score, and confusion matrices were calculated to evaluate model performance. Receiver operating characteristic (ROC) curves and the corresponding area under the curve (AUC) were additionally used to assess discriminative ability (25). For the binary classification task, agreement between model predictions and the expert reference standard was further evaluated using Cohen's kappa coefficient (26, 27).

To assess the anatomical plausibility of model decision-making, gradient-weighted class activation mapping (Grad-CAM) was applied to visualize feature activation in the final convolutional layer of the network (22). Heatmap overlays were generated for cases in which model predictions were concordant with the reference labels. Two spine surgeons with experience in scoliosis management independently reviewed the Grad-CAM results to determine whether the model's attention was primarily focused on the iliac apophysis and related anatomical structures relevant to Risser staging.

### 2.8 Clinical reader study design and evaluation

To evaluate the potential clinical utility of the AI system within a real-world reading workflow, an independent clinical reader study was conducted to compare reader performance and efficiency with and without AI assistance.

#### 2.8.1 Participants

Three spine surgeons participated in the reader study, including one senior spine surgeon with 10 years of experience and two junior spine surgeons with 5 and 3 years of experience, respectively. These readers were not involved in establishing the expert consensus reference standard and were blinded to the consensus labels during both reading sessions.

#### 2.8.2 Cases

A total of 50 standing posteroanterior full-spine digital radiographs were randomly sampled from the independent test set using stratified sampling to preserve the distribution of Risser 0–2 and Risser 3–5 cases. All readings were performed on the original full-spine radiographs to closely reflect routine clinical follow-up conditions.

#### 2.8.3 Reading protocol

A two-session crossover design was used. In the first session, all readers independently performed binary classification (Risser 0–2 vs. 3–5) without access to AI outputs. After a washout period of at least 7 days to minimize recall bias, a second reading session was conducted in which readers were provided with the AI-generated binary classification results and the corresponding Grad-CAM heatmaps as explanatory references. Although the AI system internally focused on the pelvic ROI, this automated processing was not visible to the readers. All final decisions were made by the readers based on the complete full-spine radiographs. Case presentation order was randomized in both sessions.

#### 2.8.4 Outcome measures

The primary outcomes were agreement between reader assessments and the expert consensus reference standard, evaluated using Cohen's kappa coefficient, and overall classification accuracy. Agreement was used to quantify concordance with the reference standard, while accuracy was reported as a descriptive performance measure. The secondary outcome was reading efficiency, defined as the time interval from image opening to confirmation of the final classification decision for each case.

#### 2.8.5 Statistical analysis

All analyses were performed using paired observations from the same reader and the same case under unaided and AI-assisted conditions. Continuous variables are presented as mean ± standard deviation unless otherwise specified. Normality of paired reading-time differences was assessed using Shapiro–Wilk tests before applying paired *t*-tests, with no significant deviation from normality detected for any reader (R1: *P* = 0.106; R2: *P* = 0.248; R3: *P* = 0.944). Reading times were therefore compared using paired *t*-tests, and mean time differences (ΔTime) were reported. Differences in classification accuracy were assessed using McNemar's test, which is appropriate for paired binary outcomes (27). Agreement between reader assessments and the expert reference standard was quantified using Cohen's kappa coefficient. Changes in kappa (*Δκ*) were estimated using case-level bootstrap resampling with 2,000 iterations to obtain 95% confidence intervals and two-sided bootstrap *P* values (28). All statistical tests were two-sided, and *P* values < 0.05 were considered statistically significant.

#### 2.8.6 Presentation of AI assistance

During the AI-assisted reading session, readers were shown the AI-predicted binary classification (Risser 0–2 vs. 3–5) together with the corresponding Grad-CAM heatmaps. To minimize potential effects of probability thresholds and confidence display on reader behavior, AI outputs were presented as categorical suggestions without mandatory probability or confidence scores. Readers were free to accept or reject the AI suggestions, and the final decisions were made independently by the readers and recorded as the outcomes for analysis.

## 3 Results

### 3.1 Baseline characteristics of the study cohort

A total of 875 standing posteroanterior full-spine digital radiographs from patients with AIS were included in this study. The dataset was divided at the patient level into a training set (*n* = 612), a validation set (*n* = 87), and an independent test set (*n* = 176).

The overall mean age of the cohort was approximately 14 years, consistent with the typical age distribution of patients presenting with AIS. Female patients accounted for 60.8% of the cohort (532/875). With respect to skeletal maturity, patients classified as Risser stages 0–2 slightly outnumbered those classified as Risser stages 3–5 (55.0% vs. 45.0%).

The distributions of age, sex, and Risser stratification were generally comparable across the training, validation, and test sets, indicating an adequate balance among the subsets (Table 1).

**Table 1:** Baseline characteristics of AIS patients in training, validation, and test sets.

Variable	Training set (*n* = 612)	Validation set (*n* = 87)	Test set (*n* = 176)	Total (*n* = 875)
Age (years), mean ± SD	13.9 ± 2.4	13.6 ± 2.5	13.7 ± 2.3	13.9 ± 2.4
Female, *n* (%)	381 (62.3%)	52 (59.8%)	99 (56.2%)	532 (60.8%)
Male, *n* (%)	231 (37.7%)	35 (40.2%)	77 (43.8%)	343 (39.2%)
Risser 0–2, *n* (%)	332 (54.2%)	55 (63.2%)	94 (53.4%)	481 (55.0%)
Risser 3–5, *n* (%)	280 (45.8%)	32 (36.8%)	82 (46.6%)	394 (45.0%)

Values are presented as mean ± SD or *n* (%).

### 3.2 Overall performance of the Risser 0–2 vs. 3–5 binary classification

In the binary classification task distinguishing Risser stages 0–2 from Risser stages 3–5, all three CNN architectures showed good discriminative performance on the validation and test sets, with test-set AUC values ranging from 0.931 to 0.944 (Table 2).

**Table 2:** Performance comparison of three CNN models (Risser 0–2 vs. 3–5).

**Model**	**Dataset**	**Accuracy**	**Precision**	**Recall**	**F1-score**	**Cohen's *κ***	**AUC**
ResNet-18	Val	0.874	0.872	0.873	0.873	0.745	0.919
ResNet-18	Test	0.875	0.833	0.875	0.874	0.748	0.938
ResNet-34	Val	0.912	0.920	0.925	0.919	0.839	0.962
ResNet-34	Test	0.847	0.846	0.849	0.846	0.693	0.931
DenseNet-121	Val	0.897	0.895	0.899	0.896	0.792	0.934
DenseNet-121	Test	0.847	0.848	0.851	0.846	0.694	0.944

Values represent classification metrics for the validation and test sets and are rounded to three decimal places.

However, the three models differed in validation-to-test stability. ResNet-34 achieved the highest validation performance, with an accuracy of 0.912, Cohen's *κ* of 0.839, and AUC of 0.962, but its test-set performance declined to an accuracy of 0.847, *κ* of 0.693, and AUC of 0.931. DenseNet-121 also showed a decline in accuracy and *κ* from validation to test, despite achieving the highest test-set AUC of 0.944. In contrast, ResNet-18 showed the most stable performance, with accuracy of 0.874 on the validation set and 0.875 on the test set, and *κ* values of 0.745 and 0.748, respectively. Therefore, ResNet-18 was selected as the primary model for subsequent analyses because it provided the most stable generalization on the independent test set. The corresponding training and validation loss curves for the three CNN architectures are provided in Supplementary Figure S2.

**Figure 3:**
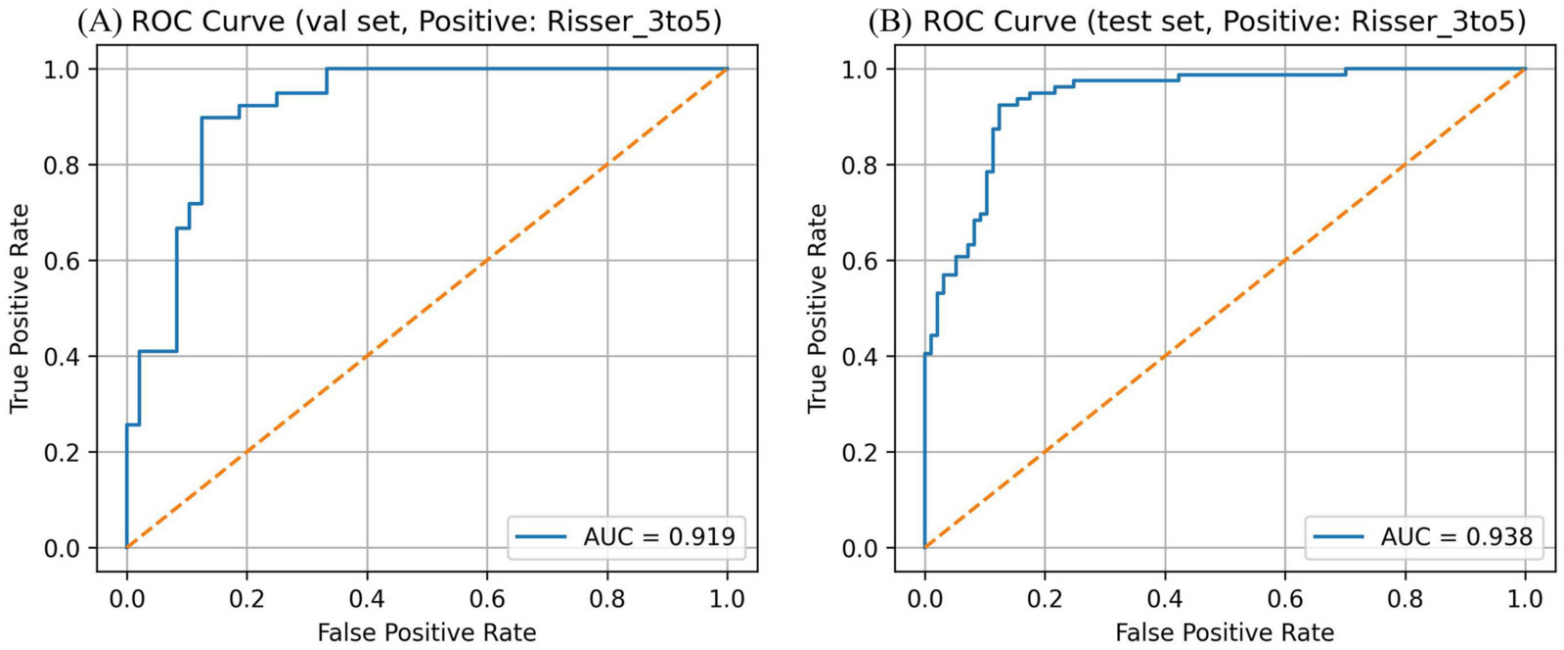
Receiver operating characteristic curves of the selected ResNet-18 model for binary Risser stratification. Receiver operating characteristic (ROC) curves are shown for the validation and independent test sets in the binary classification task distinguishing Risser stages 0–2 from Risser stages 3–5. The x-axis represents the false positive rate, and the y-axis represents the true positive rate. The colored curves indicate model performance in each dataset, and the corresponding area under the curve (AUC) values are shown in the legend. The diagonal dashed line represents the reference line for random classification.

**Figure 4:**
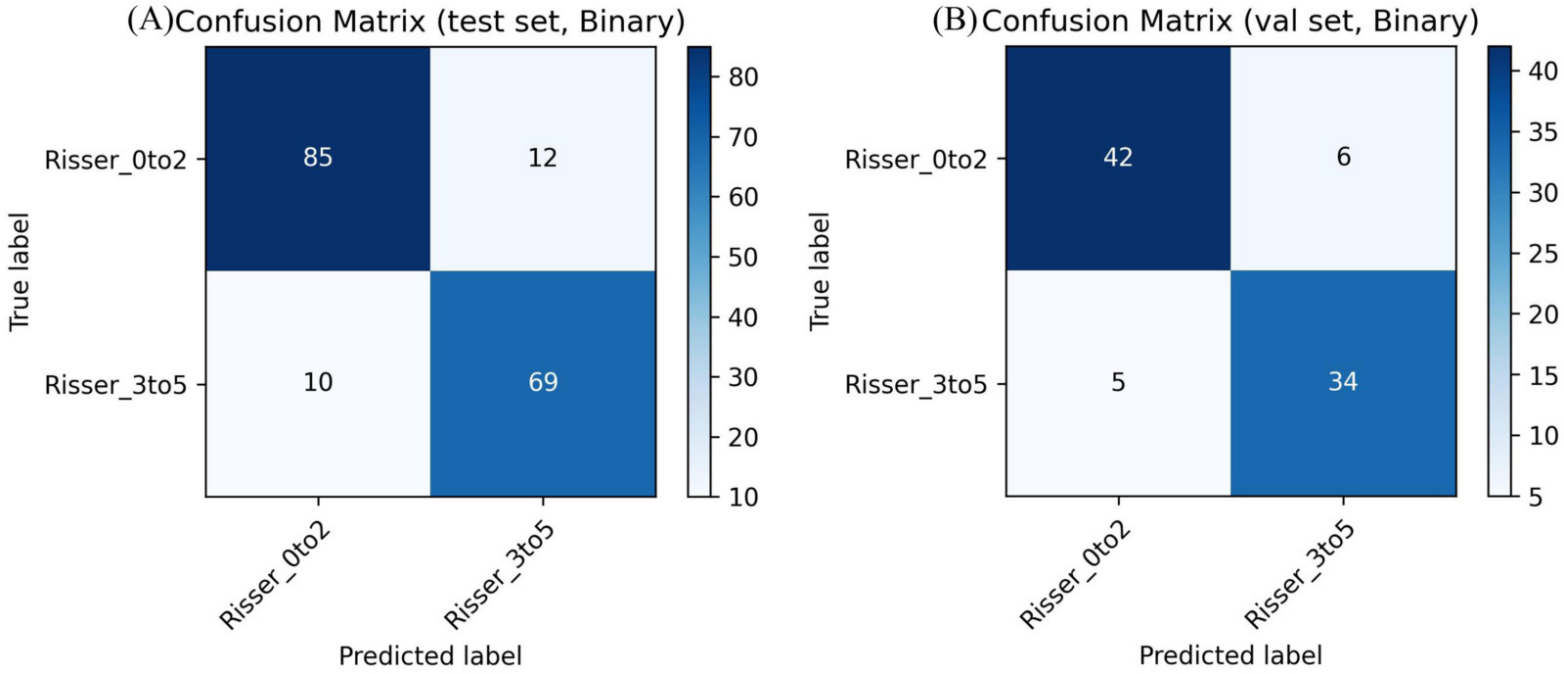
Confusion matrices of the selected ResNet-18 model for binary Risser stratification. Confusion matrices are shown for the validation and independent test sets in the binary classification task distinguishing Risser stages 0–2 from Risser stages 3–5. Rows indicate the expert consensus reference labels, and columns indicate the model-predicted labels. Diagonal cells represent correctly classified cases, whereas off-diagonal cells represent misclassified cases. Darker colors indicate a larger number of cases in the corresponding cell.

ROC curves confirmed good discrimination by the selected ResNet-18 model in both the validation and test sets, with AUC values of 0.919 and 0.938, respectively (Figure 3). The confusion matrices further showed balanced classification performance for the Risser 0–2 and Risser 3–5 groups, with most errors occurring near the clinical decision boundary between the two maturity groups (Figure 4).

### 3.3 Clinical reader study results

Under the unaided condition, the three readers showed different levels of performance in the binary Risser classification task. Using the expert consensus as the reference standard, the senior spine surgeon achieved an accuracy of 86.0% with a Cohen's *κ* of 0.710. The two junior surgeons achieved accuracies of 78.0% and 72.0%, with corresponding *κ* values of 0.550 and 0.430, respectively (Table 3).

**Table 3:** Reader performance with and without AI assistance.

Reader	Time without AI, s	Time with AI, s	*Δ*Time, s	*P* value	Accuracy without AI, %	Accuracy with AI, %	*P* value	κ without AI	κ with AI
R1	34.7 ± 3.7	25.0 ± 1.8	−9.7	<0.001	86.0	88.0	1.000	0.710	0.750
R2	43.2 ± 3.1	31.0 ± 2.0	−11.8	<0.001	78.0	88.0	0.125	0.550	0.750
R3	42.6 ± 3.2	31.8 ± 2.1	−11.3	<0.001	72.0	86.0	0.039	0.430	0.710

Values for reading time are presented as mean ± standard deviation. Each reader assessed 50 cases. R1 was a senior spine surgeon with 10 years of experience; R2 and R3 were junior spine surgeons with 5 and 3 years of experience, respectively.

ΔTime was calculated as reading time with AI minus reading time without AI; negative values indicate shorter reading time with AI assistance. The first *P* value column refers to paired t-tests for reading time, and the second *P* value column refers to McNemar's tests for accuracy. κ indicates Cohen's kappa coefficient against the expert consensus reference standard.

With AI assistance, classification performance improved across all readers. The senior surgeon's accuracy increased to 88.0%, with *κ* increasing to 0.750. For the two junior surgeons, accuracies increased to 88.0% and 86.0%, with *κ* values increasing to 0.750 and 0.710, respectively.

Reading efficiency also improved significantly with AI assistance. Compared with unaided reading, the mean reading time decreased from 34.7 ± 3.7 s to 25.0 ± 1.8 s for the senior surgeon, corresponding to a reduction of 9.7 s per case. For the two junior surgeons, the mean reading time decreased from 43.2 ± 3.1 s to 31.0 ± 2.0 s and from 42.6 ± 3.2 s to 31.8 ± 2.1 s, corresponding to reductions of 11.8 and 11.3 s per case, respectively. All paired comparisons for reading time were statistically significant (all *P* < 0.001).

Overall, AI assistance consistently reduced reading time across all readers, while the magnitude of improvement in accuracy and agreement varied by reader experience level. Detailed paired comparisons and bootstrap-derived confidence intervals for changes in *κ* are provided in Table 3 and Supplementary Table S2. Reader–AI concordance under the assisted condition is summarized in Supplementary Table S4.

### 3.4 Exploratory analysis of six-class Risser staging

As an exploratory analysis, we evaluated model performance for the original six-class Risser staging task (stages 0–5). Overall performance for six-class classification was substantially lower than that observed for the binary task. Misclassifications were predominantly concentrated between adjacent stages (e.g., stages 1–2, 2–3, and 3–4), indicating the inherent difficulty of fine-grained discrimination under routine standing full-spine radiographic conditions.

Detailed performance metrics and confusion matrices for the six-class classification are provided in the Supplementary Materials (Supplementary Table S3 and Supplementary Figure S3).

### 3.5 Grad-CAM–based interpretability analysis

**Figure 5:**
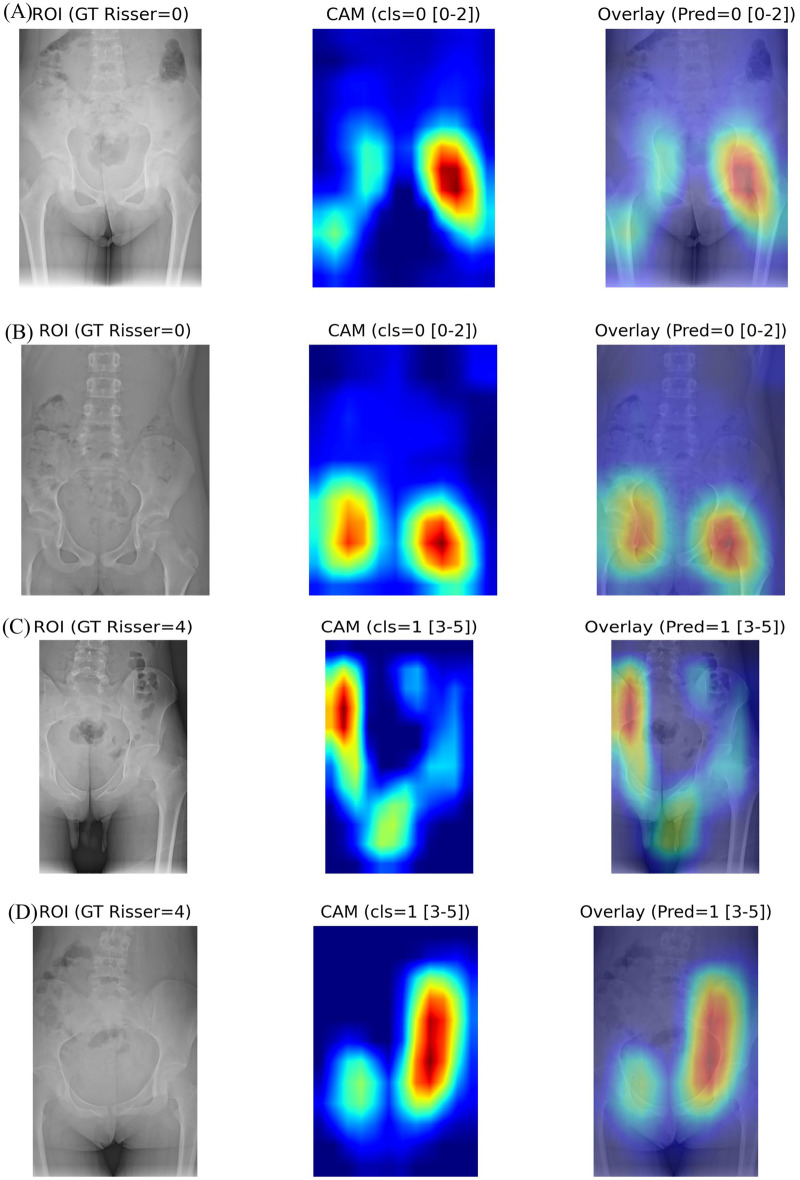
Representative Grad-CAM visualizations of the selected ResNet-18 model. Representative gradient-weighted class activation mapping (Grad-CAM) images are shown to illustrate the anatomical regions contributing to the model's binary Risser classification decisions. For each case, the left panel shows the cropped pelvic ROI used as model input, the middle panel shows the Grad-CAM heatmap, and the right panel shows the heatmap overlaid on the ROI image. Warmer colors, including yellow and red, indicate regions with greater contribution to the model prediction, whereas cooler colors indicate lower contribution. The visualizations show that the model primarily focused on the iliac apophysis and adjacent ossification regions, which are anatomically relevant for Risser staging.

Using ResNet-18 as an example, Grad-CAM visualizations were generated for representative correctly classified cases from the test set (Figure 5). For cases classified as Risser 0–2, high-response regions were primarily localized to the bilateral iliac wings and the lateral margins of the iliac crest, corresponding to the anatomical regions typically assessed by clinicians when determining the onset of iliac apophyseal ossification (Figures 5A,B).

For cases classified as Risser 3–5, Grad-CAM visualizations showed prominent attention over the continuous ossification band along the superior aspect of the iliac crest and its posterior extension, which aligns with the anatomical features evaluated during manual assessment of apophyseal maturation and fusion (Figures 5C,D).

Additional Grad-CAM visualizations from a broader range of patients, including selected misclassified cases, are provided in Supplementary Figure S4.

Overall, the Grad-CAM results indicate that model predictions were primarily driven by morphological and density changes in the iliac apophysis and adjacent iliac crest, rather than by the femoral heads, soft tissues, or other unrelated structures. These findings support the anatomical plausibility of the model's decision-making process in the context of clinical Risser assessment.

## 4 Discussion

In this study, we developed and evaluated a decision-oriented deep-learning model for automated skeletal maturity stratification using standing full-spine digital radiographs from 875 patients with AIS. The primary model (ResNet-18) performed well in the binary classification of Risser stages 0–2 vs. 3–5 on the independent test set, with substantial agreement with the expert consensus reference standard. Grad-CAM analyses further showed that model predictions were mainly driven by anatomically relevant regions, including the iliac apophysis and adjacent ossification bands, rather than by unrelated image structures. By focusing on the clinically meaningful dichotomy of “skeletally immature vs. near or fully mature” rather than fine-grained six-stage classification, the proposed approach is more closely aligned with key decision points in AIS management, including follow-up scheduling, brace treatment, and surgical timing.

The clinical reader study further supports the potential role of the proposed system as a decision-support tool in routine reading workflows. Rather than replacing clinician judgment, the model appeared to provide a useful second reference, particularly for less experienced readers, by improving consistency with the expert reference standard and reducing the time needed to reach a binary maturity decision. This finding is clinically relevant because Risser assessment on full-spine radiographs can be affected by the small size of the iliac apophysis, variable image quality, overlapping structures, and reader experience.

Compared with prior automated Risser assessment studies based on dedicated pelvic radiographs, abdominal radiographs, or EOS imaging, the main practical advantage of the present approach is its use of routine standing full-spine radiographs that are already acquired during AIS follow-up. Direct numerical comparison across studies should be interpreted cautiously because imaging modalities, class definitions, reference standards, and validation designs differ. Nevertheless, the present workflow may be easier to integrate into routine AIS surveillance because it does not require additional imaging, a separate acquisition protocol, or specialized imaging equipment. The incomplete agreement between readers and AI outputs, as further summarized in Supplementary Table S4, also supports the use of the model within a human-in-the-loop workflow, in which AI assistance serves as an interpretable reference rather than an autonomous decision-maker (29).

Most prior studies on automated Risser assessment have been developed using dedicated pelvic radiographs, abdominal radiographs, or imaging acquired with specialized systems such as EOS (18, 19, 21). Although high performance has been reported under these controlled imaging conditions, translation to routine follow-up settings remains challenging in centers where standing full-spine digital radiographs constitute the standard imaging modality. Practical barriers include additional equipment requirements, workflow disruption, and concerns regarding extra radiation exposure.

In contrast, the present study leverages standing posteroanterior full-spine radiographs that are routinely acquired at nearly every outpatient visit for AIS follow-up (18, 19, 21). By applying an automated pelvic region-of-interest extraction strategy, skeletal maturity stratification can be achieved without additional imaging, protocol modification, or increased radiation dose. This design enables seamless integration into existing clinical workflows and supports the use of the proposed model as a standardized decision-support output during routine AIS surveillance.

From a task-design perspective, most prior studies have treated Risser staging as a six-class classification problem, focusing primarily on discriminating between individual stages and optimizing overall agreement. However, in real-world clinical decision-making, the distinction between Risser 0–2 and 3–5 is often more critical than fine-grained differentiation between adjacent stages such as 1 vs. 2 or 3 vs. 4 (5, 6). Lower Risser stages (0–2) generally indicate substantial remaining growth potential and a higher risk of curve progression, necessitating closer surveillance and brace intervention. In contrast, higher stages (3–5) suggest that skeletal growth is near completion or has ceased, with a markedly reduced risk of progression, thereby informing brace adjustment or discontinuation and more definitive surgical timing.

Driven by this clinical rationale, the present study defined binary stratification (Risser 0–2 vs. 3–5) as the primary endpoint to align model outputs with the core decision points in routine AIS management. Six-class Risser classification was evaluated only as an exploratory analysis. The observed misclassifications were predominantly concentrated between adjacent stages, highlighting the inherent subjectivity and blurred boundaries of fine-grained staging when using routine standing full-spine radiographs. These findings further support that a clinically decision-oriented binary stratification may offer greater reproducibility and practical value than detailed multi-class staging in real-world follow-up settings.

With respect to model selection, the comparison across candidate architectures showed that stronger validation-set performance did not necessarily translate into better test-set generalization. ResNet-34 achieved the highest validation metrics, but showed the largest decline on the independent test set, particularly in Cohen's *κ*. DenseNet-121 showed a similar, although less pronounced, validation-to-test decline in agreement. This pattern suggests weaker generalization stability and possible overfitting in the higher-capacity models, especially in the setting of a moderate-sized, single-center medical imaging dataset. In contrast, ResNet-18 showed the most consistent validation and test performance while maintaining high discriminative ability. For this reason, ResNet-18 was selected as the primary model, prioritizing generalization stability and practical deployability over the highest validation-set score.

The inherent subjectivity of Risser staging and the associated interobserver variability have long posed challenges in clinical management. Previous studies have reported that interobserver agreement for manual Risser assessment is generally only moderate, and discrepancies of multiple stages may occur between readers. These inconsistencies are partly attributable to indistinct iliac apophyseal boundaries, variable exposure conditions, pelvic rotation, and overlap from bowel gas or soft tissues, and also reflect the intrinsic “gray zones” in radiographic interpretation of apophyseal ossification onset and fusion.

In the present study, the model showed substantial agreement with the expert consensus reference standard for the clinically important binary stratification of Risser 0–2 vs. 3–5. This finding suggests that the model may help standardize the assessment of whether meaningful growth potential remains. When considered alongside the results of the clinical reader study, the results support the role of AI assistance as a standardized second reference that may improve reading efficiency and reduce inter-reader variability, without replacing physician judgment.

In addition, this study used an expert consensus labeling workflow involving multiple spine surgeons. Initial disagreements were resolved through adjudication and discussion, which helped reduce the subjective variability inherent in conventional Risser assessment. This consensus-based reference standard provided a more consistent basis for model training and agreement evaluation. However, expert consensus should be viewed as a way to reduce reference-label variability, not as a method for eliminating it completely. Some uncertainty is still unavoidable in borderline cases, particularly between adjacent Risser stages. This residual ambiguity may partly explain why the exploratory six-class task performed less well than the binary Risser 0–2 vs. 3–5 task.

Model interpretability is also a prerequisite for clinical adoption. Using Grad-CAM–based visualization, we examined the regions driving model predictions. In correctly classified cases, high-activation areas were predominantly localized to the bilateral iliac apophysis and adjacent ossification zones, which closely correspond to the anatomical regions emphasized during clinical Risser assessment. In selected misclassified cases, attention shifts were observed, suggesting that automated assessment may be affected by suboptimal visualization of the iliac apophysis. Potential contributors include exposure variation, pelvic rotation or obliquity, severe scoliosis-related pelvic asymmetry, and overlap from bowel gas or soft tissues. These factors may reduce the clarity of the iliac apophyseal margin and make the Risser 0–2 vs. 3–5 boundary more difficult to classify. These findings highlight the importance of structured image-quality assessment and careful data curation in future refinements of the model.

Several limitations of this study should be acknowledged. First, this was a single-center retrospective study, with all images acquired from the same geographic region and using a single digital radiography system. External validation in multicenter cohorts will therefore be necessary to further assess the generalizability of the proposed model (30, 31). In addition, skeletal maturation may vary by sex and ethnic background. Although sex distribution was reported in the cohort, this study was not designed or powered to evaluate sex-specific model performance, and ethnicity-specific information was not available in a structured form for subgroup validation. The external validity of the model across different populations should therefore be interpreted with caution. Future studies should include multicenter and more demographically diverse cohorts, with prespecified sex-stratified and population-specific performance analyses.

Second, the rule-based ROI strategy has inherent limitations. Although the crop was designed and checked to include the bilateral iliac crests, a fixed rectangular ROI may still be affected by marked pelvic rotation, pelvic obliquity, or severe scoliosis-related asymmetry. Direct resizing of the rectangular ROI to a square input may also have introduced mild geometric distortion because aspect-ratio-preserving padding was not used. The present task focused on categorical Risser stratification rather than quantitative pelvic morphometry, but future studies should compare this pragmatic preprocessing approach with padding-based aspect-ratio-preserving resizing, landmark-guided cropping, or segmentation-based pelvic localization.

Third, we were unable to perform a formal subgroup analysis of misclassified cases according to exposure quality, pelvic rotation, or obesity, because these variables were not consistently recorded in a structured form in the retrospective dataset. In particular, BMI or other body-habitus measures were not reliably available for all patients. Future studies should prospectively collect structured image-quality ratings, pelvic-positioning measures, and anthropometric information to allow prespecified error analyses.

Fourth, the clinical reader study was conducted at a single center with a limited number of readers and was restricted to a binary classification task (30). Future studies involving a larger number of readers with varying levels of experience, as well as more complex and realistic clinical workflows, are warranted to better evaluate robustness and clinical applicability.

Fifth, this study did not prospectively assess the direct impact of the AI system on clinical decision-making, such as follow-up interval selection, brace prescription adjustment, or surgical timing. The potential influence of AI-assisted Risser stratification on these downstream clinical decisions remains to be explored in future prospective studies.

Future work should also compare the proposed approach with other skeletal maturity systems, particularly Sanders staging (32). Because Sanders staging usually requires hand radiographs, a direct comparison was not possible in the present retrospective cohort of full-spine radiographs. Future studies should also evaluate hybrid multimodal maturity models that combine imaging findings with clinical variables such as sex, chronological age, growth velocity, menarchal status when applicable, and other skeletal maturity indicators. These models may provide a more complete estimate of remaining growth and improve decision support in AIS management.

## 5 Conclusion

In conclusion, this interpretable deep-learning model provides a clinically actionable, radiation-sparing solution for skeletal maturity stratification directly from routine standing full-spine radiographs. By focusing on the critical therapeutic threshold of Risser 0–2 vs. 3–5, this anatomically transparent tool integrates seamlessly into real-world workflows, where it significantly enhances reading efficiency and diagnostic consistency, particularly among less experienced clinicians. Ultimately, deploying this human-in-the-loop system standardizes the assessment of remaining growth potential, offering a highly scalable framework to optimize risk stratification and guide timely interventions in the routine management of adolescent idiopathic scoliosis.

## Data Availability

The original contributions presented in the study are included in the article/Supplementary Material, further inquiries can be directed to the corresponding author/s.
