# Supplementary material for: From routine full-spine radiographs to decision-oriented Risser stratification: an interpretable deep-learning approach
Source: Front Pediatr. 2026 Jun 17;14:1832066. doi: 10.3389/fped.2026.1832066 (PMC13318898; doi:10.3389/fped.2026.1832066)
Supplement: Supplementary file 1 [file Supplementaryfile1.docx]

**Supplementary Materials**


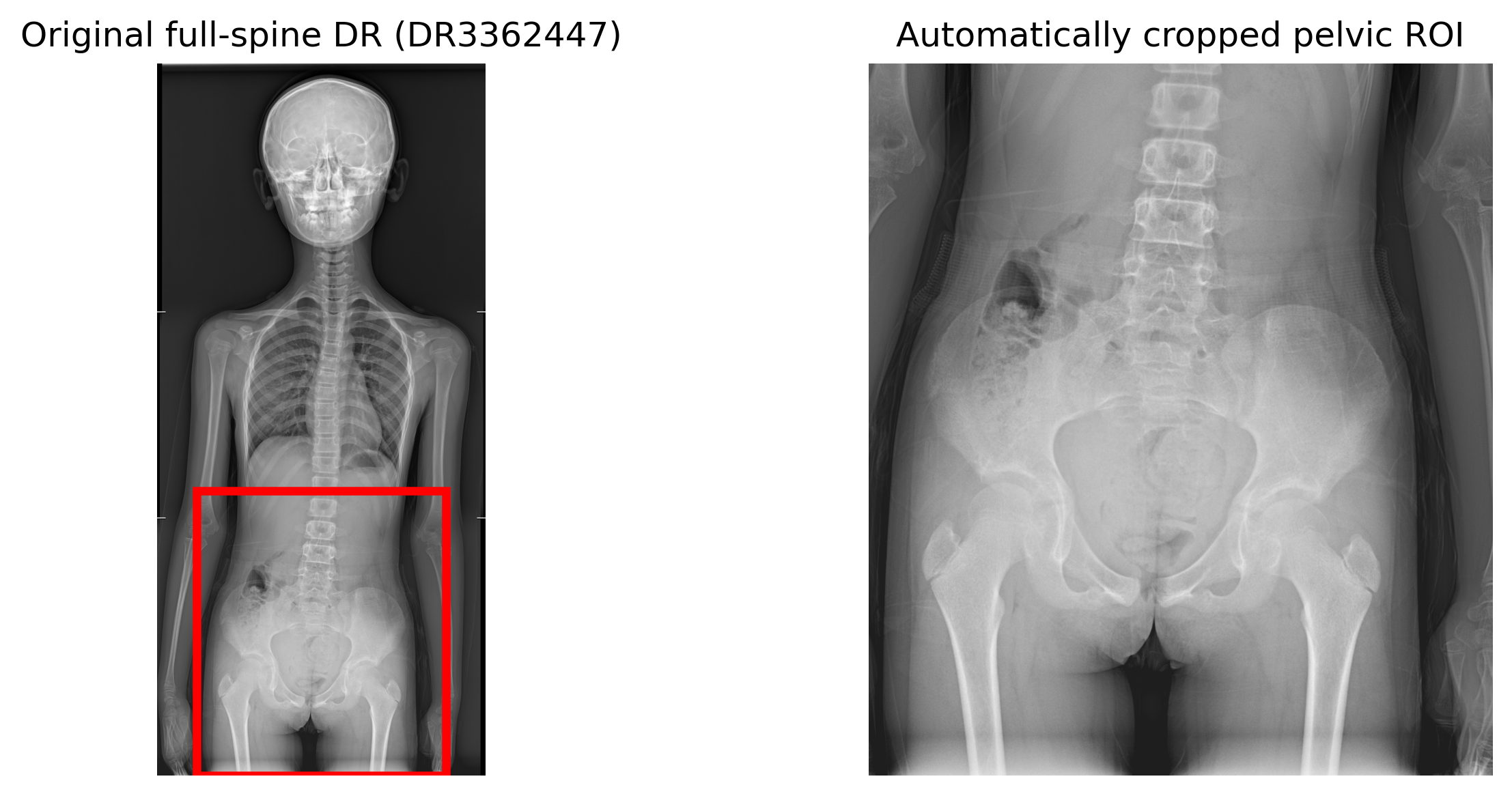


**Supplementary Figure S1. Illustration of pelvic region-of-interest (ROI) extraction.**

The left panel shows an example of an original standing full-spine anteroposterior radiograph, with the predefined pelvic ROI highlighted for illustration. The right panel shows the corresponding automatically cropped pelvic ROI used as input for model training and inference. This ROI was designed to consistently cover the iliac wings and iliac apophysis, which are anatomically relevant for Risser staging. For clarity, ROI cropping was applied exclusively within the AI model pipeline. In the clinical reader study, all readers independently assessed the original full-spine radiographs without ROI cropping, in order to reflect routine clinical reading conditions.


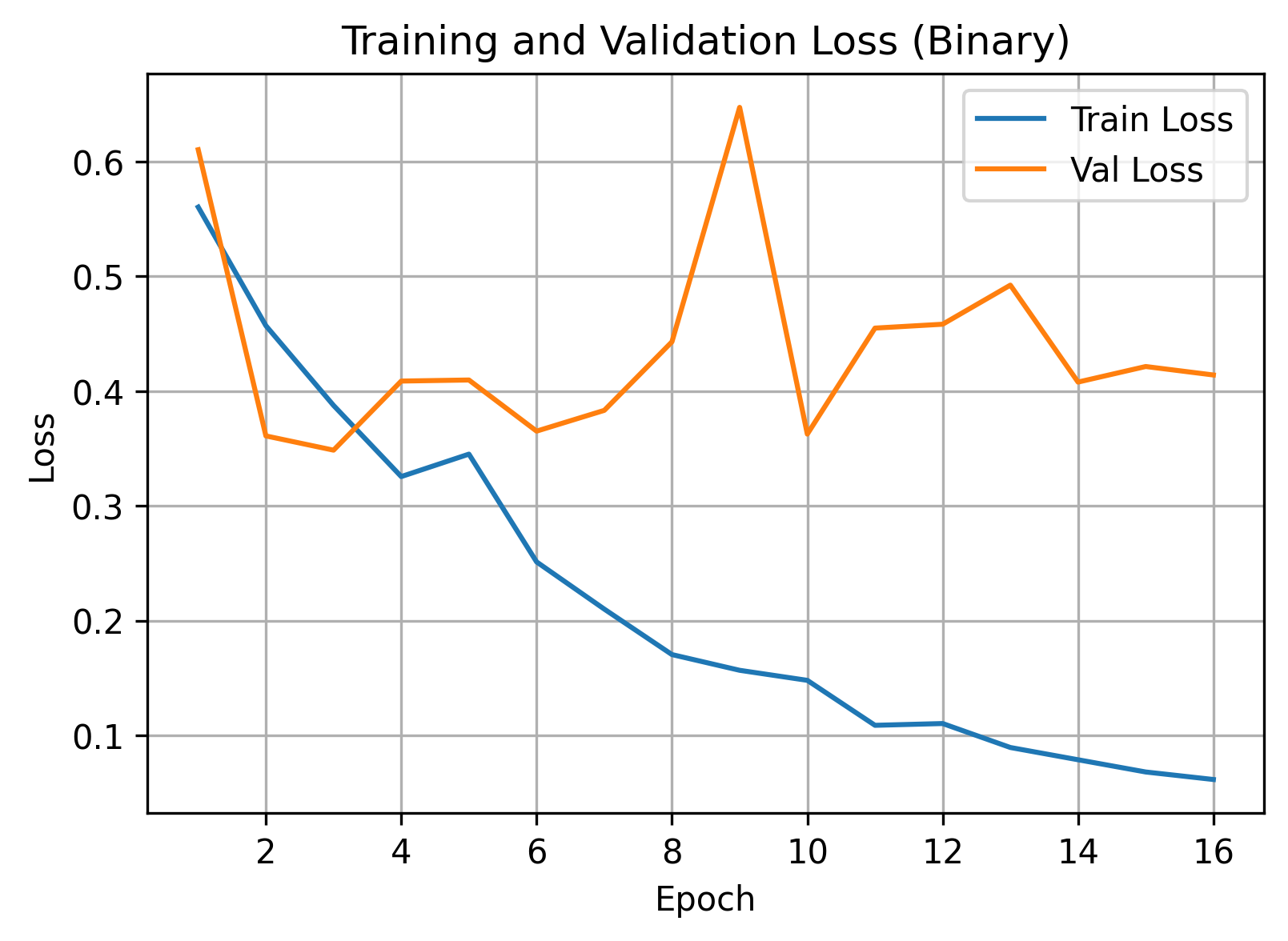

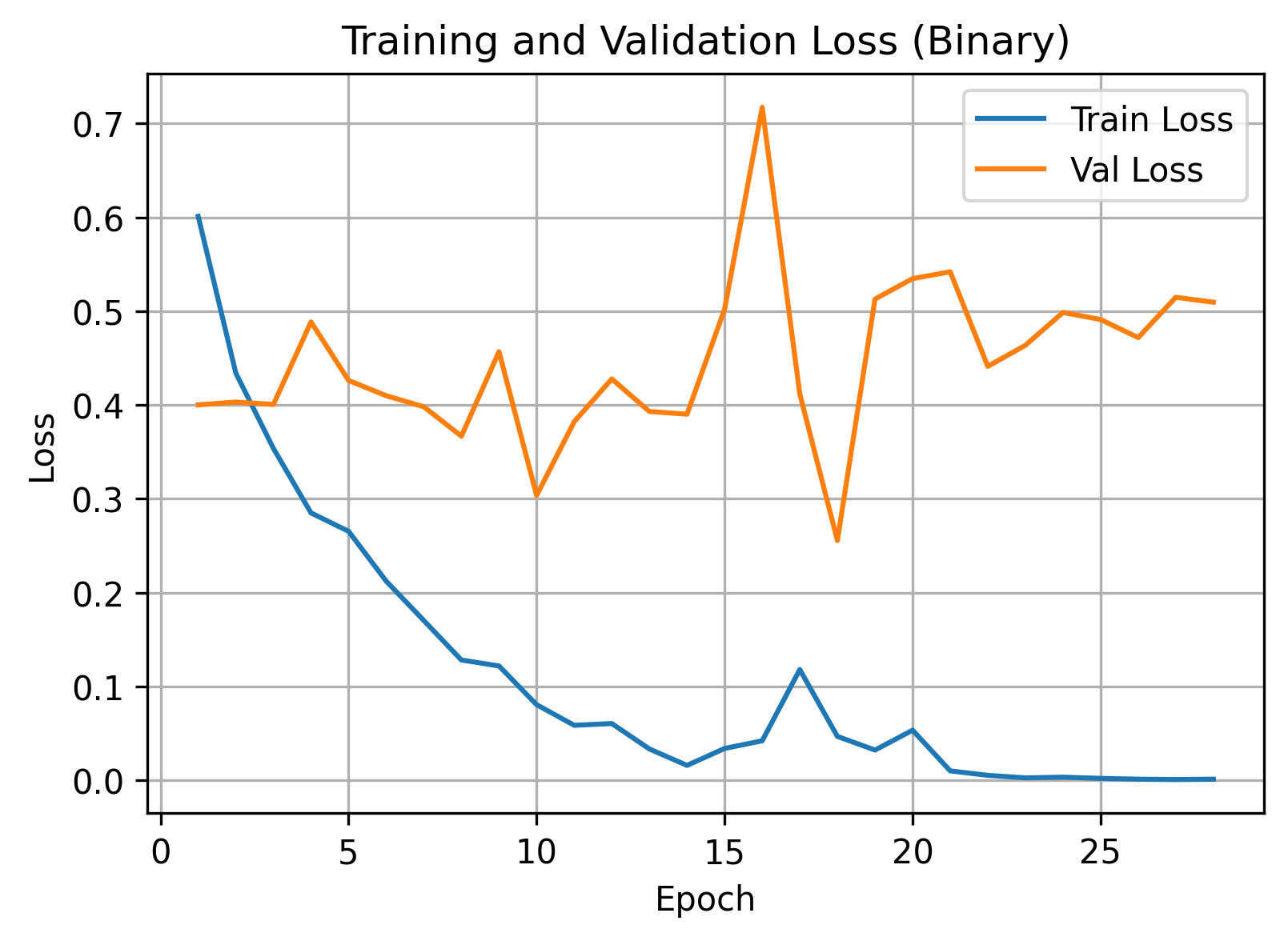

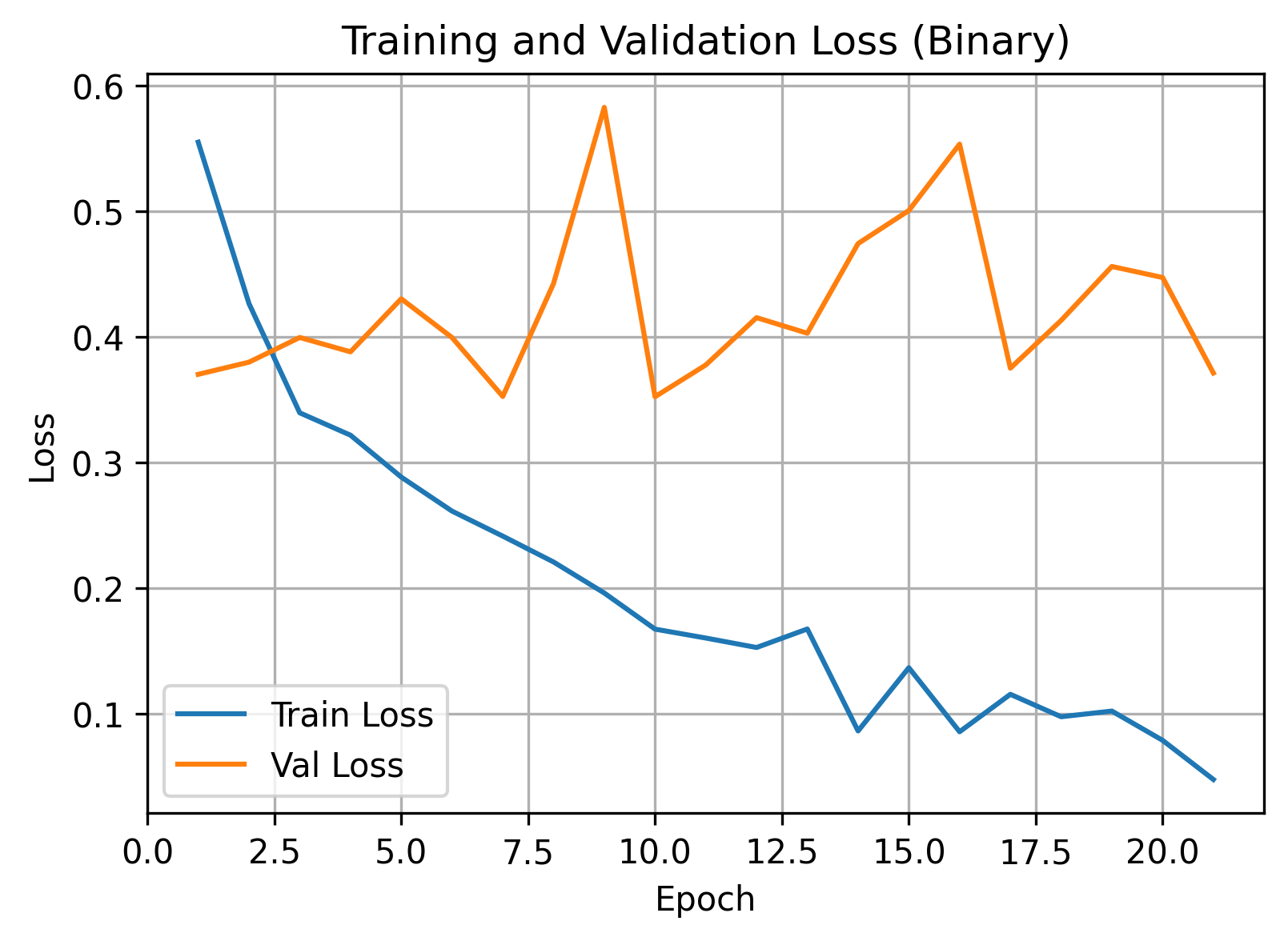


**Supplementary Figure S2. Training and validation loss curves of three CNN architectures for binary Risser classification.**

**(A) ResNet-18, (B) ResNet-34, and (C) DenseNet-121 training–validation loss trajectories.**All three models were trained under the same data split, preprocessing pipeline, and training protocols. ResNet-18 shows the most stable convergence with minimal fluctuation in validation loss, whereas ResNet-34 and DenseNet-121 exhibit greater variability, reflecting increased model capacity and sensitivity to limited medical imaging data. These curves support the selection of ResNet-18 as the primary model in the main study.


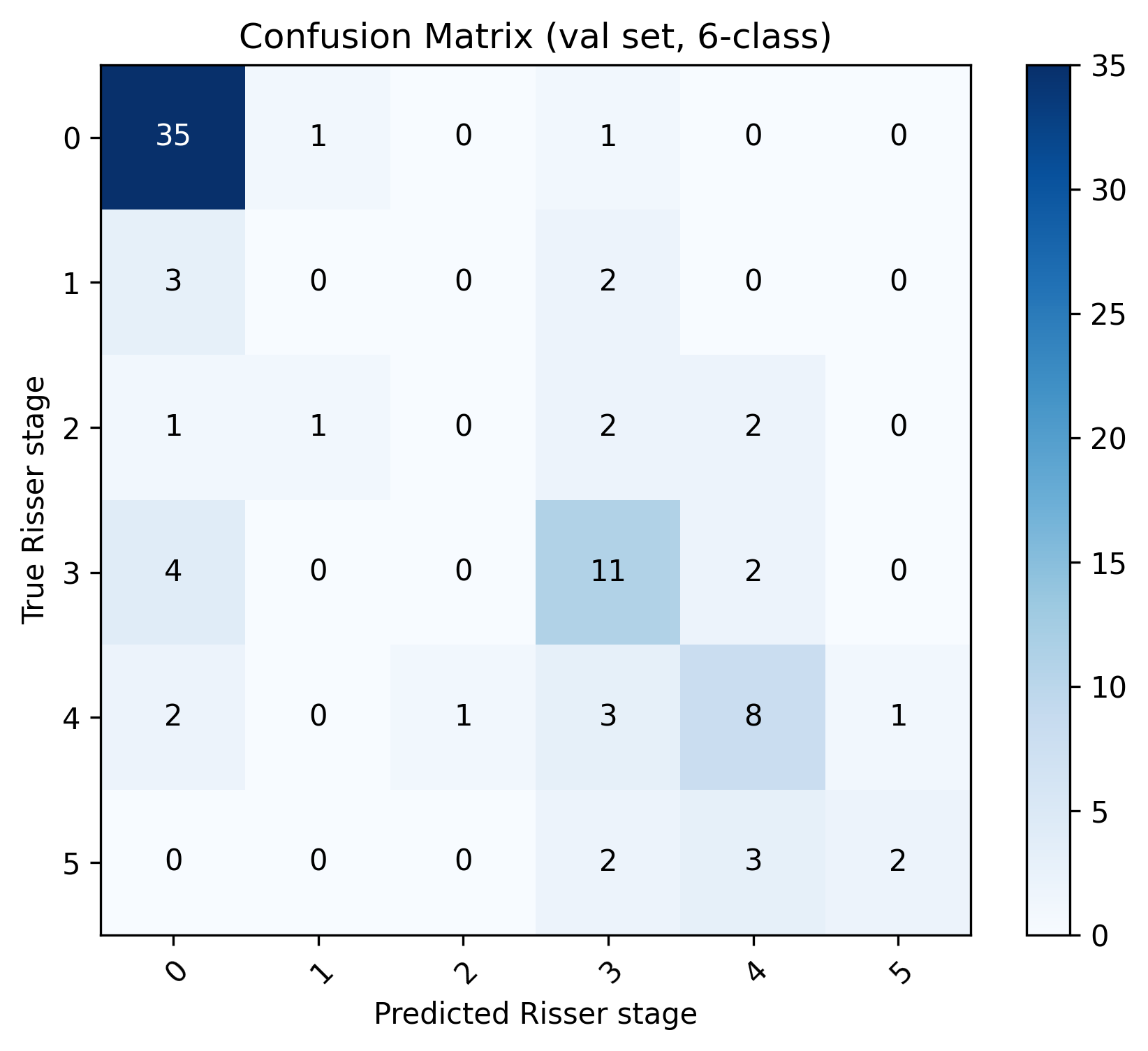


(A)


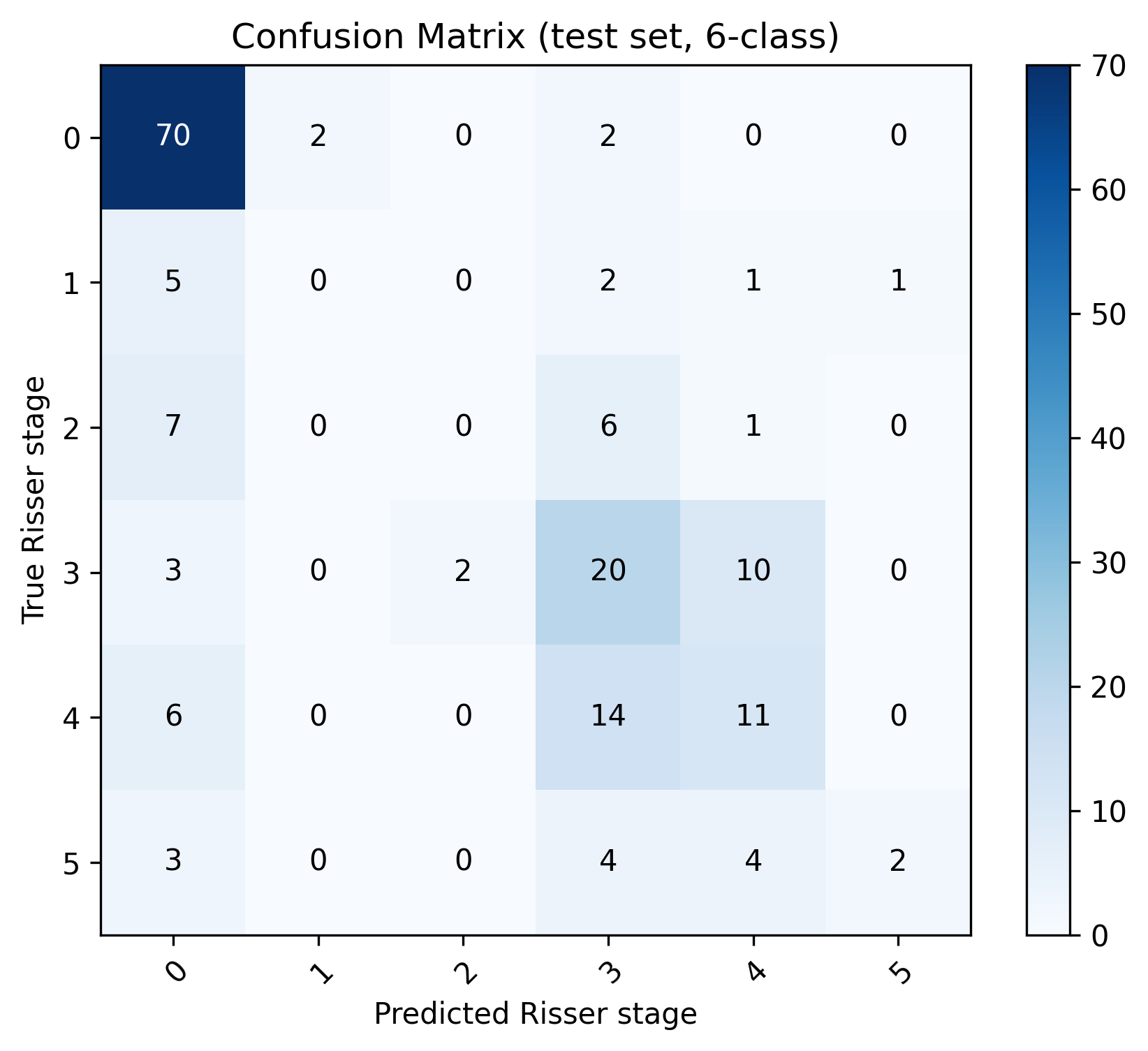


(B)

**Supplementary Figure S3. Confusion matrices for the six-class Risser classification task (exploratory analysis).**

(A) Confusion matrix for the validation set, and (B) confusion matrix for the independent test set using the six-class Risser model (Risser 0–5). The matrices illustrate the distribution of model predictions across true Risser stages, highlighting correct classifications along the diagonal and typical misclassification patterns between adjacent stages. These matrices are provided for exploratory analysis only and are not intended for primary clinical interpretation.


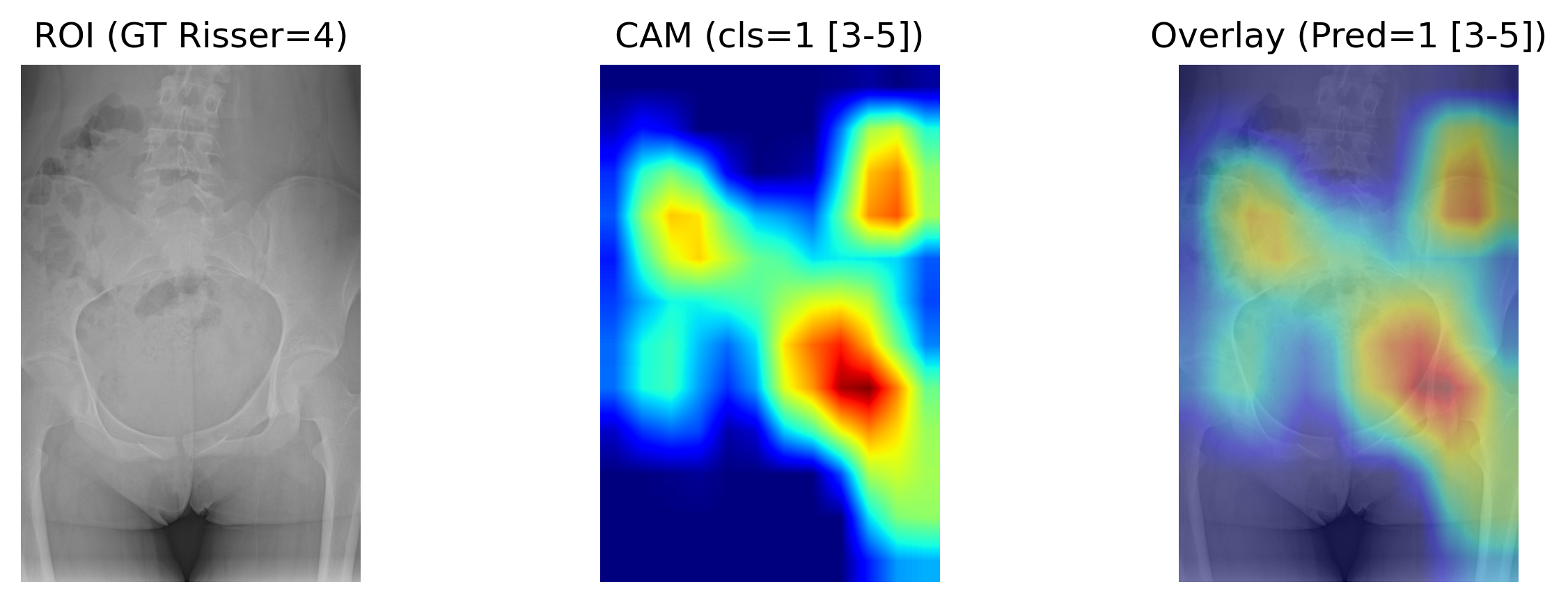


(F)


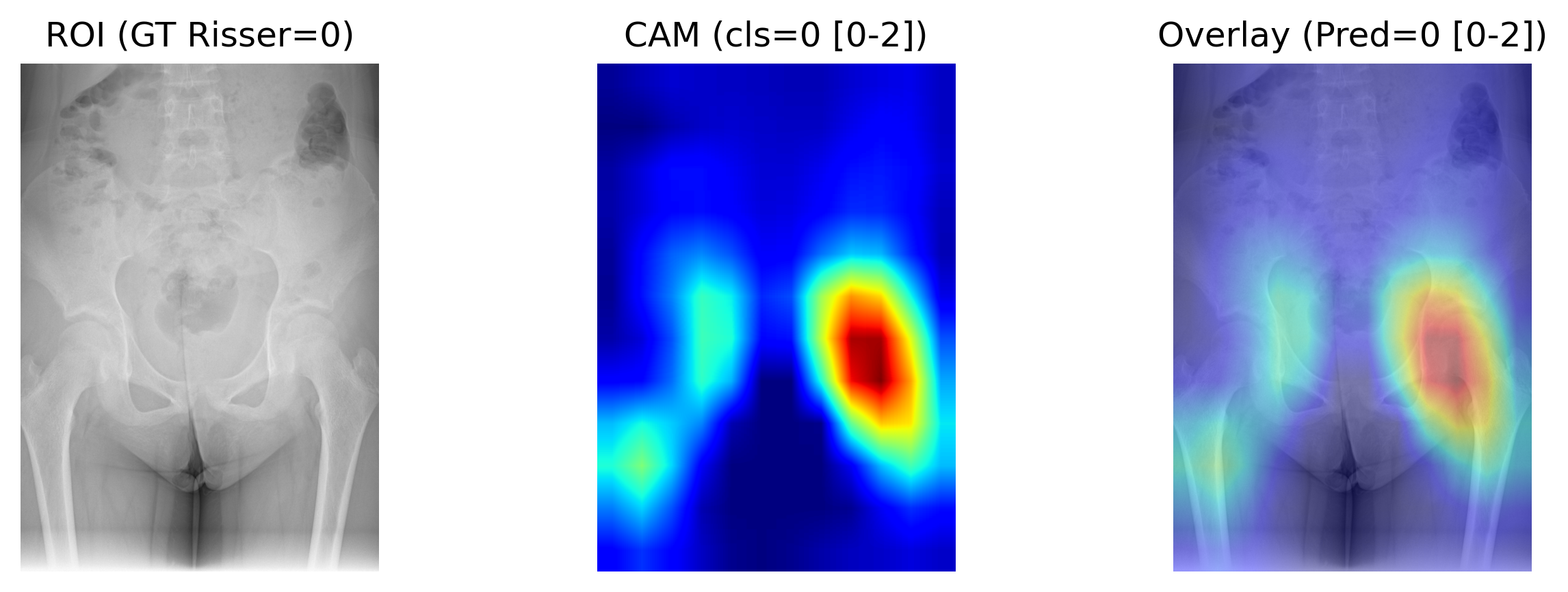

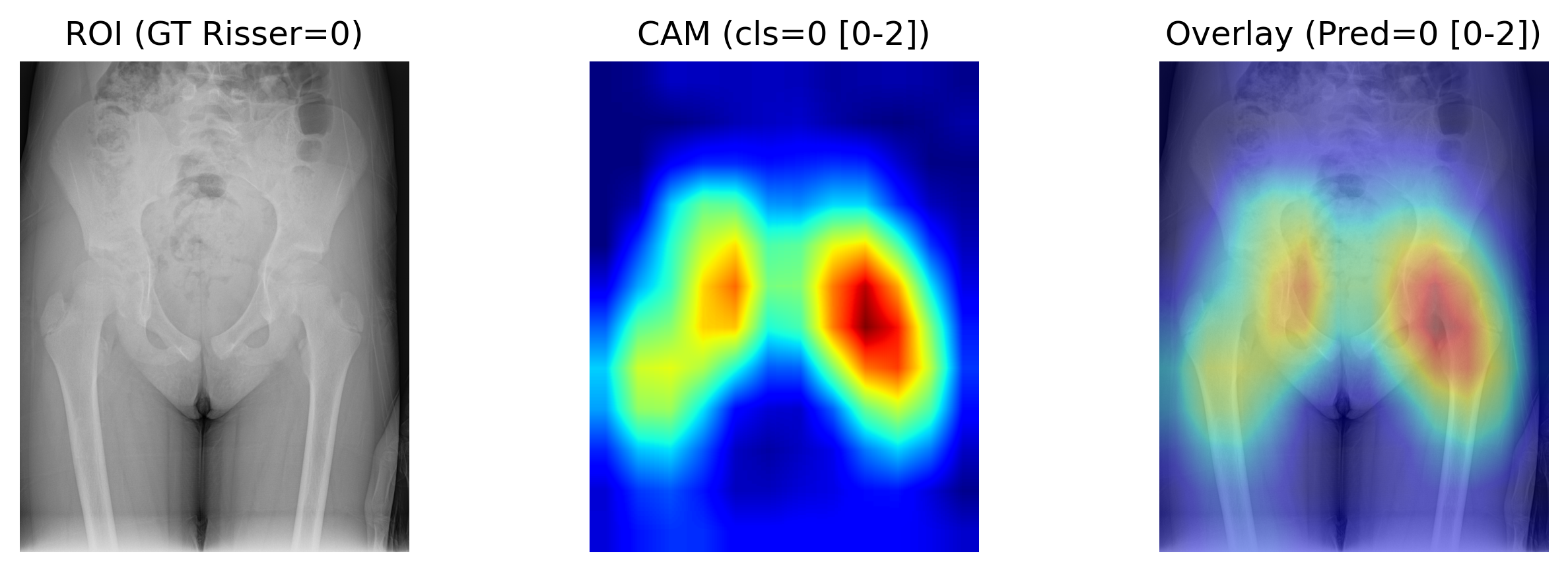


(A)


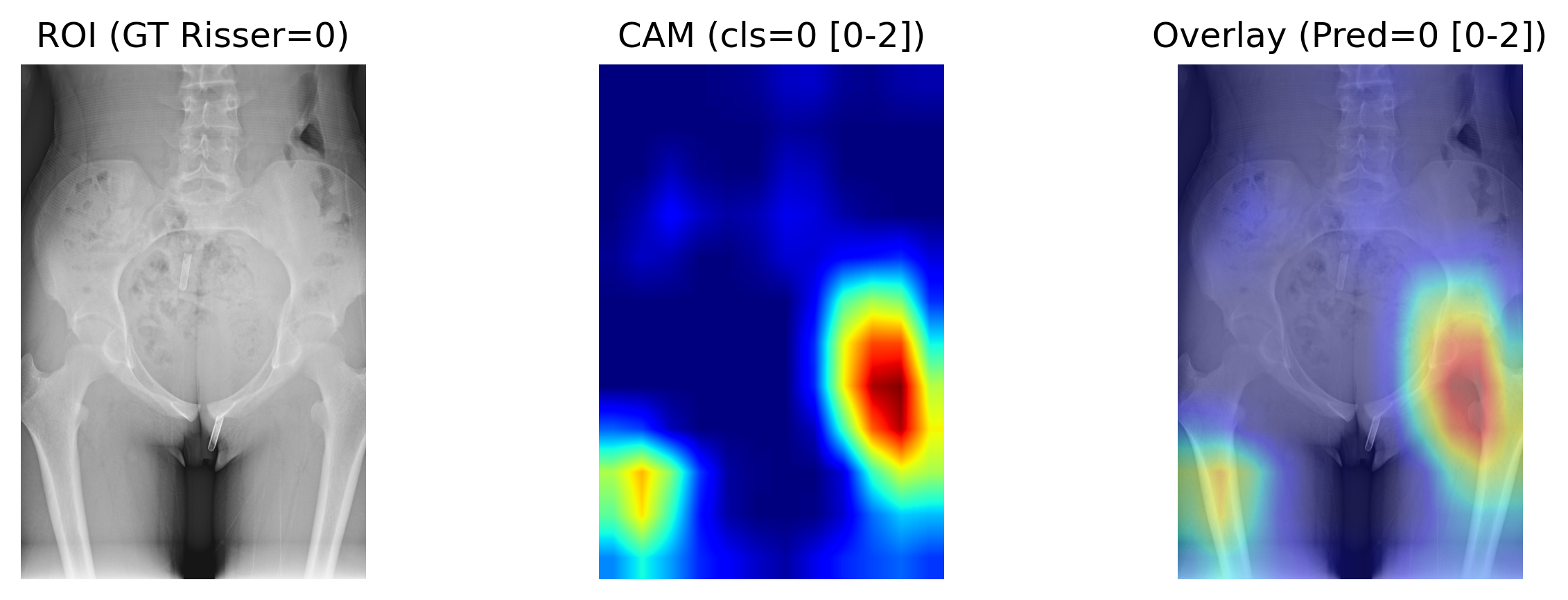


(D)


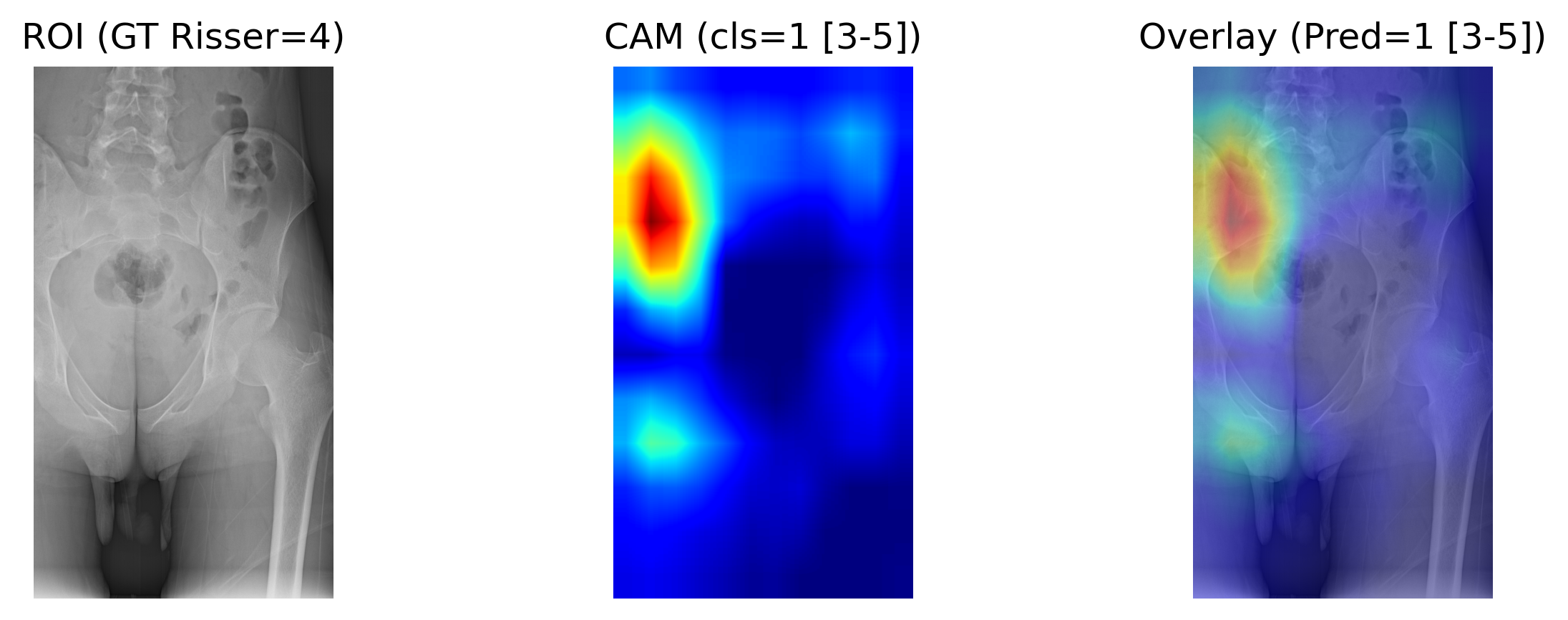


(E)


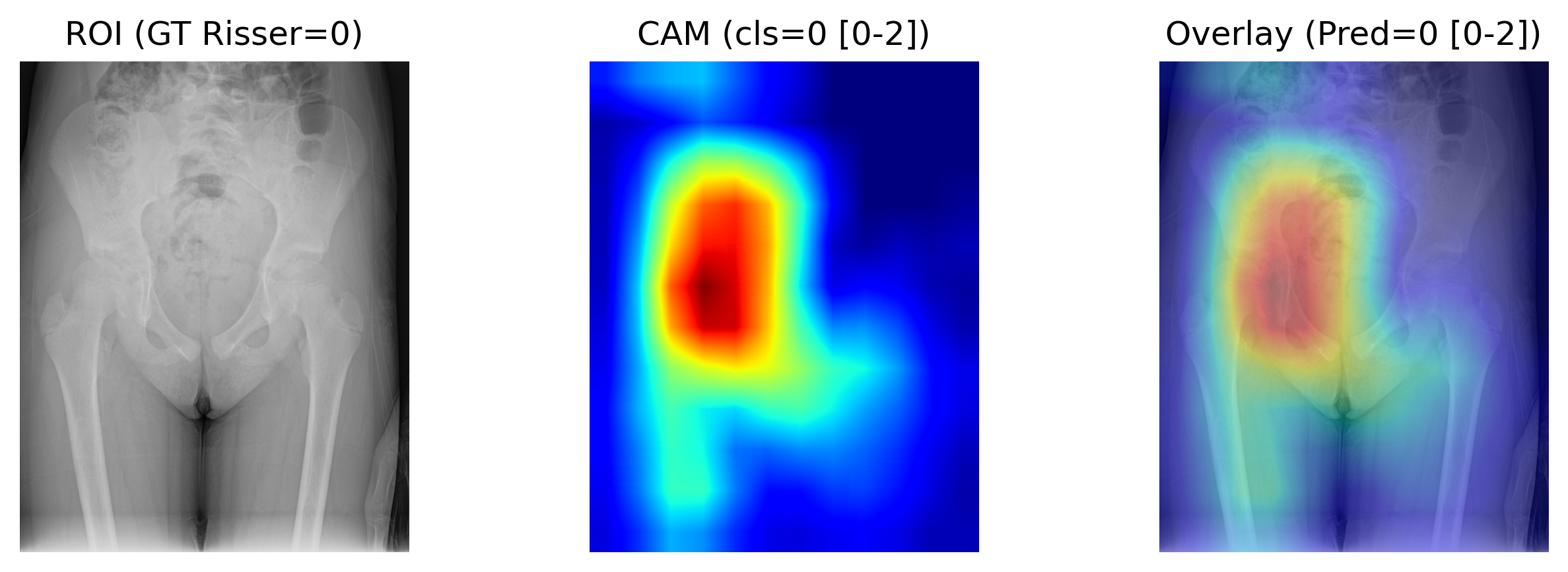


(B)

(C)


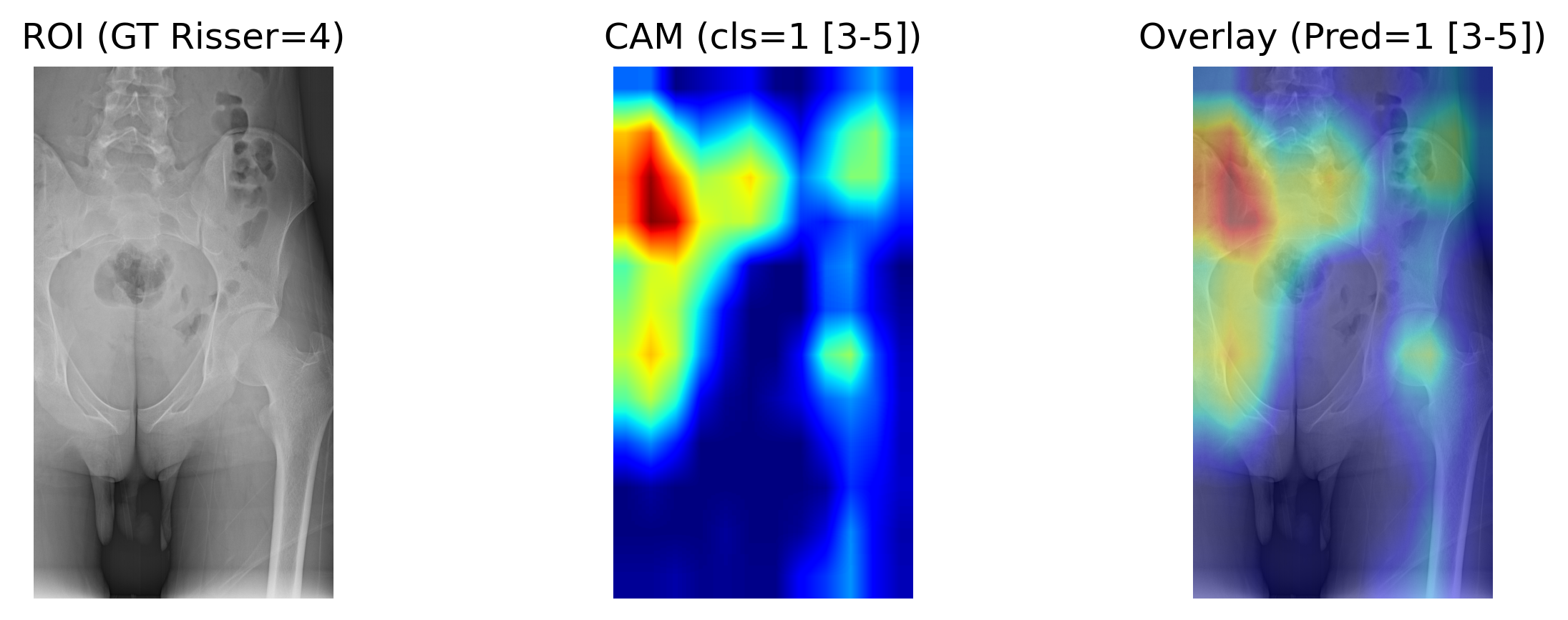


(G)


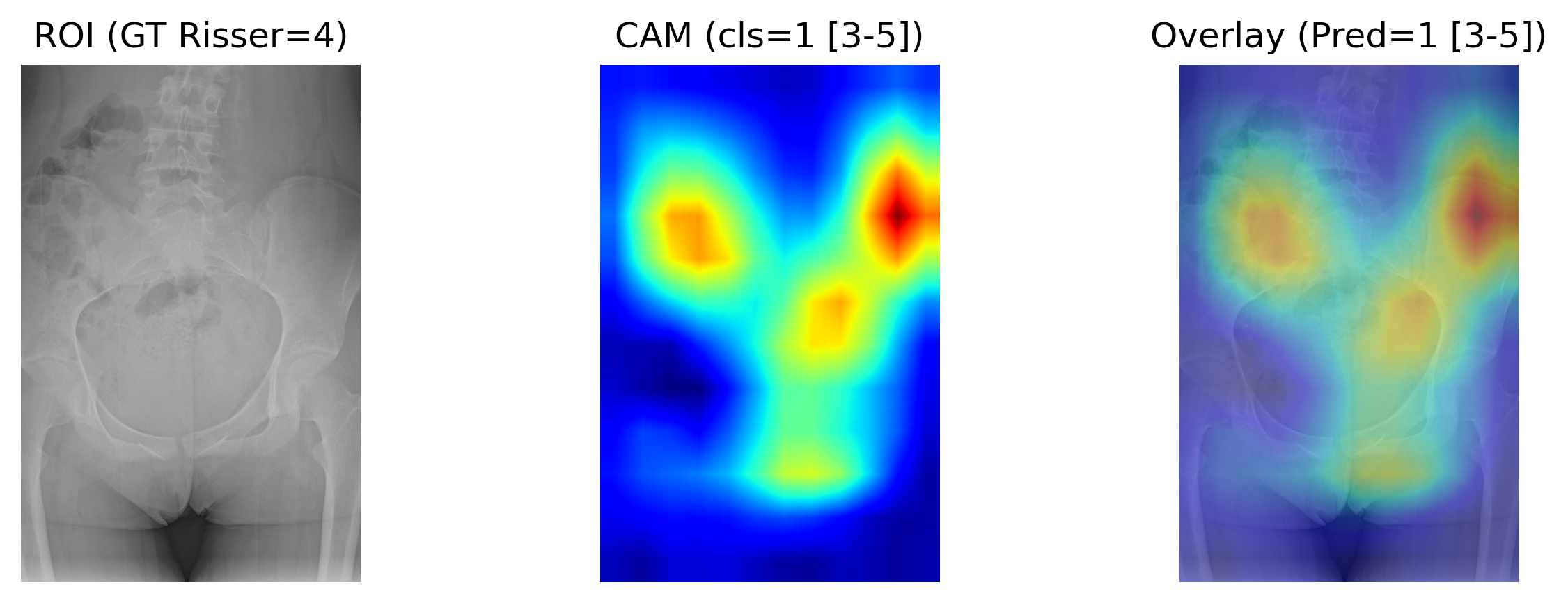


(H)


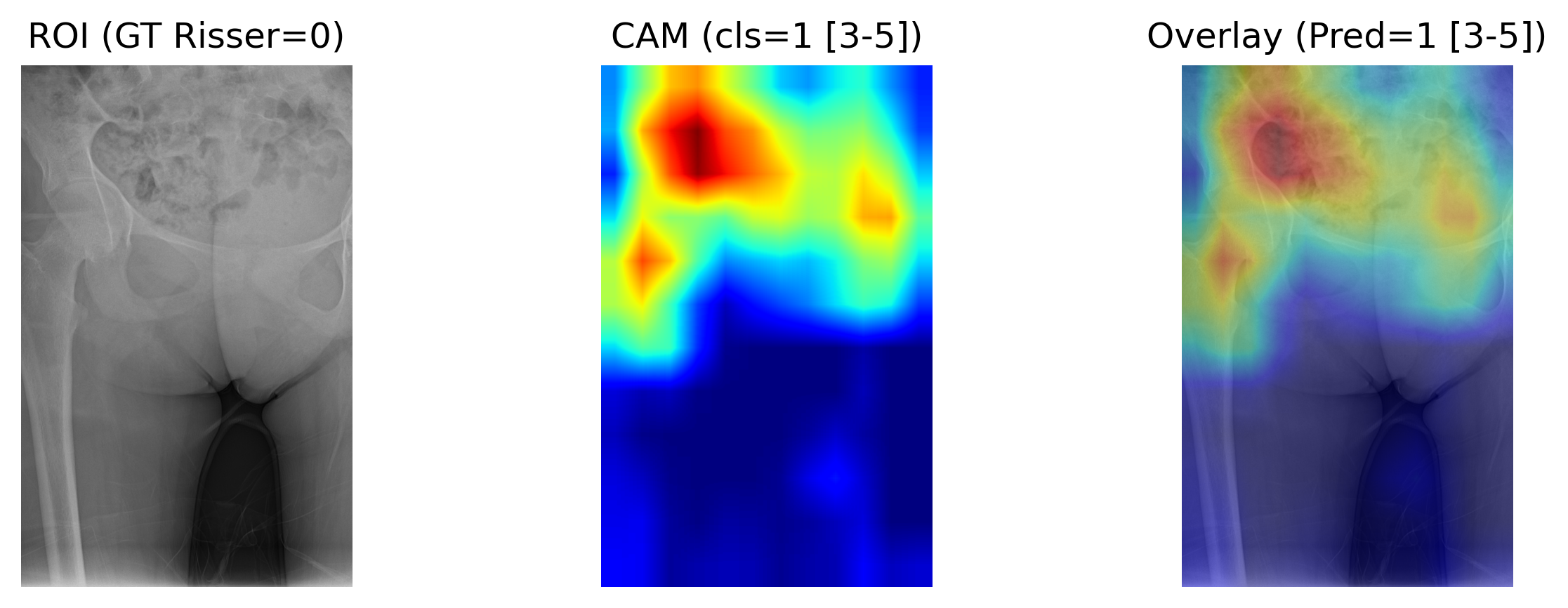


(I)


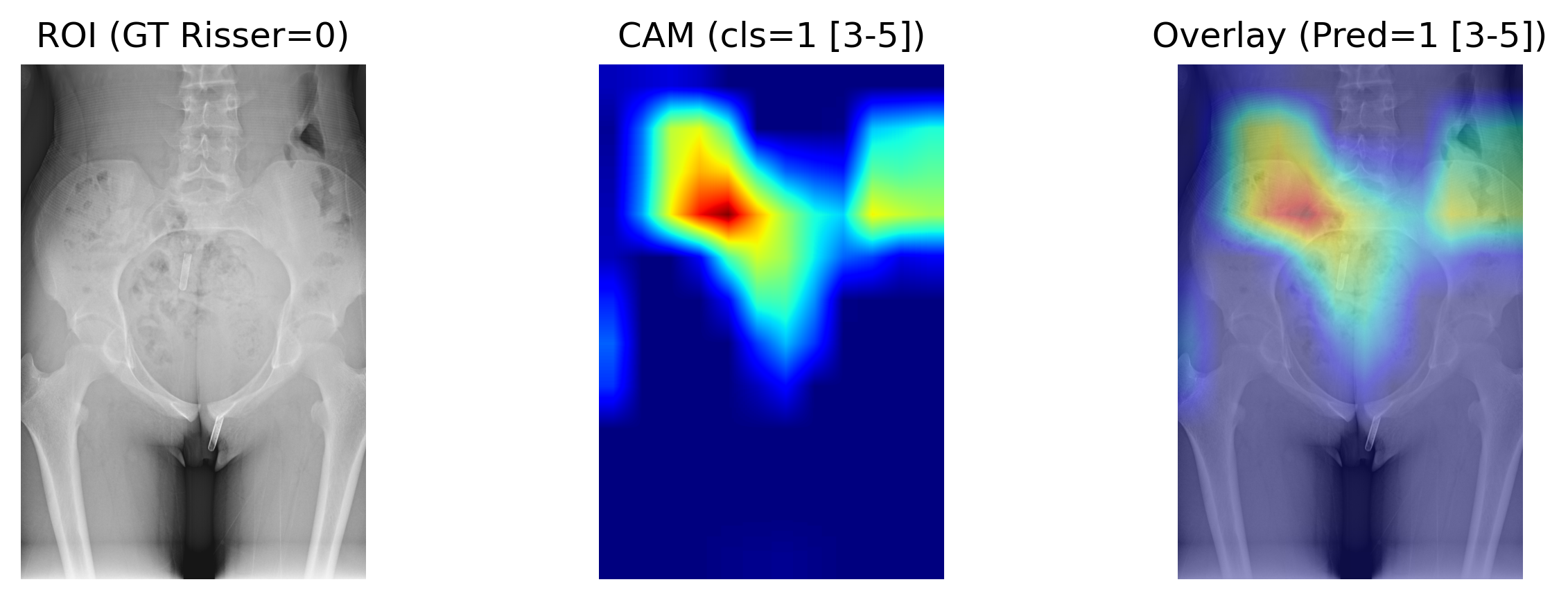


(J)

**Supplementary Figure S4. Additional Grad-CAM visualizations demonstrating model focus on the iliac apophysis across different patients.**

Additional representative Grad-CAM visualizations illustrating how the binary classification model (Risser 0–2 vs. 3–5) attends to the iliac apophysis region across diverse cases. For each patient example, three panels are shown: 1. ROI input (automatically cropped pelvic region), 2. Grad-CAM heatmap, 3. Overlay of the heatmap on the ROI. The visual patterns demonstrate that the model consistently allocates high attention to the iliac crest apophysis, aligning with the anatomical basis of Risser grading. These cases include both correctly and incorrectly predicted samples, highlighting model behavior under varying stages and morphological appearances. Grad-CAM visualizations are generated from the model input to illustrate internal decision focus and are independent of the clinical reader study setup.

**Supplementary Table S1. Training configuration used for model development and final ResNet-18 evaluation.**

| **Item** | **Setting** |
| --- | --- |
| Backbone architecture | ResNet-18 (pre-trained on ImageNet; final selected model) |
| Task | Binary classification (Risser 0–2 vs. 3–5) |
| Input image size | 384×384 (predefined pelvic ROI extracted from full-spine radiographs for model input) |
| Batch size | 4 |
| Optimizer | Adam |
| Initial learning rate | 1 × 10⁻⁴ |
| Loss function | Cross-entropy loss |
| Maximum epochs | 50 |
| Early stopping | Patience = 10 epochs (no improvement in validation accuracy) |
| Train/Val/Test split | 70% / 10% / 20% (patient-level, stratified) |
| Data augmentation (training only) | Random rotation (±7°); random affine (translation ±2%, scale 0.95–1.05); brightness/contrast jitter ±0.1 |
| Normalization | ImageNet mean/std (0.485, 0.456, 0.406 / 0.229, 0.224, 0.225; used to match pretrained backbone requirements) |
| ROI extraction rule | Middle 60% width + bottom 35% height |
| Hardware setup | GPU-based training environment (PyTorch framework) |
| Model selection | Best model selected by highest validation accuracy |
| Evaluation metrics | Accuracy, Cohen’s κ, precision, recall, F1-score, ROC–AUC |

**Abbreviations:** ROI, region of interest; ROC–AUC, area under the receiver operating characteristic curve.

**Supplementary Table S2. Changes in reader performance with AI assistance.**

| **Reader** | **ΔTime (s)** | **P(time)** | **ΔAccuracy (%)** | **P(acc)** | **Δκ** | **95% CI** | **P(bootstrap)** |
| --- | --- | --- | --- | --- | --- | --- | --- |
| **R1** | −9.7 | <0.001 | +2.0 | 1.000 | +0.040 | [−0.090, 0.180] | 0.770 |
| **R2** | −11.8 | <0.001 | +10.0 | 0.125 | +0.200 | [0.000, 0.410] | 0.073 |
| **R3** | −11.3 | <0.001 | +14.0 | 0.039 | +0.280 | [0.070, 0.520] | 0.011 |

ΔTime and ΔAccuracy represent the differences between AI-assisted and unaided readings for the same reader on the same cases. P(time) was calculated using paired t-tests. P(acc) was calculated using McNemar’s test for paired binary outcomes. Δκ indicates the change in Cohen’s kappa coefficient between AI-assisted and unaided readings against the expert consensus. The 95% confidence intervals (CI) for Δκ were estimated using case-level bootstrap resampling with 2,000 iterations. P(bootstrap) denotes the two-sided bootstrap-derived P value for Δκ.

**Supplementary Table S3. Exploratory performance metrics for the six-class Risser task.**

Precision, recall, and F1-score are reported for each Risser stage in the validation and test sets. Macro- and weighted-averaged metrics are provided to account for substantial class imbalance.

Notably, the intermediate Risser stages (Risser 1–2) demonstrate markedly reduced performance, which is consistent with the known difficulty and limited interobserver reliability of fine-grained Risser staging on routine full-spine radiographs.

These findings highlight the inherent instability of six-class Risser classification in real-world clinical imaging and further justify the focus of this study on the clinically actionable binary classification (Risser 0–2 vs. 3–5) used for growth potential assessment and treatment decision-making.

**Validation Set**

| **Class** | **Support** | **Precision** | **Recall** | **F1-score** |
| --- | --- | --- | --- | --- |
| **Risser 0** | 37 | 0.778 | 0.946 | 0.854 |
| **Risser 1** | 5 | 0.000 | 0.000 | 0.000 |
| **Risser 2** | 6 | 0.000 | 0.000 | 0.000 |
| **Risser 3** | 17 | 0.524 | 0.647 | 0.579 |
| **Risser 4** | 15 | 0.533 | 0.533 | 0.533 |
| **Risser 5** | 7 | 0.667 | 0.286 | 0.400 |
| **Macro average** | 87 | 0.417 | 0.402 | 0.394 |
| **Weighted average** | 87 | 0.579 | 0.644 | 0.600 |

**Test Set**

| **Class** | **Support** | **Precision** | **Recall** | **F1-score** |
| --- | --- | --- | --- | --- |
| **Risser 0** | 74 | 0.745 | 0.946 | 0.833 |
| **Risser 1** | 9 | 0.000 | 0.000 | 0.000 |
| **Risser 2** | 14 | 0.000 | 0.000 | 0.000 |
| **Risser 3** | 35 | 0.417 | 0.571 | 0.482 |
| **Risser 4** | 31 | 0.407 | 0.355 | 0.379 |
| **Risser 5** | 13 | 0.667 | 0.154 | 0.250 |
| **Macro average** | 176 | 0.373 | 0.338 | 0.324 |
| **Weighted average** | 176 | 0.517 | 0.585 | 0.531 |

# Supplementary Table S4. Reader Agreement with AI Predictions

| **Reader** | **Level** | **Agreement with AI (%)** |
| --- | --- | --- |
| **R1** | Senior | 90.0 |
| **R2** | Junior | 89.0 |
| **R3** | Junior | 88.0 |

Values are presented as percentages per reader based on 50 cases each. Agreement indicates the proportion of cases in which the reader’s final classification was concordant with the AI output under AI-assisted conditions. This table is intended to characterize reader–AI interaction and should not be interpreted as diagnostic accuracy.
